# Mode and Tempo of Microsatellite Evolution across 300 Million Years of Insect Evolution

**DOI:** 10.3390/genes11080945

**Published:** 2020-08-16

**Authors:** Michelle Jonika, Johnathan Lo, Heath Blackmon

**Affiliations:** 1 Department of Biology, Texas A & M University, College Station, TX 77843, USA; michelle19@tamu.edu (M.J.); jl6794a@tamu.edu (J.L.); 2 Genetics Interdisciplinary Program, Texas A & M University, College Station, TX 77843, USA

**Keywords:** microsatellite evolution, insects, repetitive DNA, chromosome evolution, genome size, centromere

## Abstract

Microsatellites are short, repetitive DNA sequences that can rapidly expand and contract due to slippage during DNA replication. Despite their impacts on transcription, genome structure, and disease, relatively little is known about the evolutionary dynamics of these short sequences across long evolutionary periods. To address this gap in our knowledge, we performed comparative analyses of 304 available insect genomes. We investigated the impact of sequence assembly methods and assembly quality on the inference of microsatellite content, and we explored the influence of chromosome type and number on the tempo and mode of microsatellite evolution across one of the most speciose clades on the planet. Diploid chromosome number had no impact on the rate of microsatellite evolution or the amount of microsatellite content in genomes. We found that centromere type (holocentric or monocentric) is not associated with a difference in the amount of microsatellite content; however, in those species with monocentric chromosomes, microsatellite content tends to evolve faster than in species with holocentric chromosomes.

## 1. Introduction

Genomes contain a variety of sequence classes, many of which are repetitive in nature. The smallest of these are microsatellites—simple sequence repeats that have a 2–6-base-pair repeating motif. Microsatellites are highly polymorphic sequences that are most commonly found within non-coding portions of the genome; however, they can also be located in regulatory or intronic regions [1,2]. The location of microsatellites within the genome can have strong impacts on their stability. For instance, microsatellites occurring in regulatory or protein-coding regions tend to be highly conserved [3,4]. Similarly, conserved noncoding microsatellites which occur in the 5′ flanking regions of some protein coding genes in plants are, as their name suggests, conserved among species, possibly for their role in gene regulation [5,6]. In contrast, microsatellites found in regions without regulatory or coding functions (e.g., intergenic and some intronic regions) are likely to have little impact on organism fitness and, thus, their frequency and distribution should reflect the underlying mutation processes [7].

Microsatellites are useful to biologists as easily accessed genetic markers, and some microsatellites have fundamental impacts on organismal functioning. Because of their relative neutrality, microsatellites are useful in inferences of population demography, as well as genetic diversity [8,9]. The large quantity and variability in microsatellites even within a species make them useful for forensics [10], kinship analysis [11], and medical profiling [12]. Microsatellites also play an important role in chromatin organization [13], DNA structure [14], and centromere and telomere function [15]. However, the most studied effect of microsatellites is on the regulation of gene activity, where microsatellites can impact transcription [16], gene expression [17], protein binding [18], and translation [19], thus leading to diseases [20].

The primary mechanism for expansion and contraction of microsatellites is slippage that occurs during DNA replication [21,22]. However, differential abundance of repeats in exonic, intronic, and intergenic regions among taxa may suggest that strand slippage alone is insufficient to explain microsatellite distribution [23]. While strand slippage can account for the expansion and contraction of microsatellite content, it cannot account for large shifts in the relative abundance of types of microsatellites in closely related species (e.g., a shift from AC repeats to TA repeats being the most common).

While previous studies focused largely on specific classes of microsatellites in one or a handful of species, few studies examined the dynamics of all microsatellite content across large clades [24,25,26]. Adams et al. showed that ray-finned fish, squamate reptiles, and mammalian genomes had higher microsatellite content than crocodilian, turtle, and avian genomes. Additionally, some lineages had unusually high rates of change in microsatellite content, providing support for multiple major shifts in the microsatellite genomic landscape. The goal of our study was to determine whether microsatellites evolve differently in different clades of insects and to evaluate the impact of chromosome number, genome size, and centromere type (i.e., holocentric and monocentric) on both the content and the rate of microsatellite evolution. Our analyses revealed that chromosome number has no impact on either content or rate of microsatellite evolution, and that centromere type has no impact on total microsatellite content. However, our study showed that different insect orders have significantly different rates, and that the rate of microsatellite evolution is different among species with monocentric and holocentric chromosomes. Additionally, we found that genome size correlates with total content and rate of microsatellite evolution.

## 2. Materials and Methods

*Sequence Data:* We downloaded all available insect genome assemblies from NCBI, ENSMBL, and Baylor HGC (accession numbers and site addresses in Table S1 (Supplementary Materials), accessed August 2018). A total of 304 genomes were available spanning 18 of the 24 insect orders. Six orders were represented by single species, while Diptera and Hymenoptera were the most frequently sequenced orders with 116 and 71 species, respectively. In all cases, the most recent assembly with no masking was downloaded (accession numbers in Table S1 (Supplementary Materials)).

*Assembly quality:* Repetitive sequences are one of the central challenges in genome assembly and, because of this, it is possible that poorly assembled genomes or genomes assembled with shorter read technology will lead to inaccurate inference of microsatellite content. We took two approaches to assess and control for this possibility. Firstly, we reanalyzed data from a survey of microsatellites across 71 vertebrates [26]. In this analysis, we categorized each genome assembly by the sequencing platform (short, Sanger, and long) and tested whether genomes in these three classes had significantly different microsatellite content. If mixed data were available for a genome assembly (e.g., long-read sequencing with short-read polishing), this was classified as long read for our categories. Secondly, we also evaluated the correlation between scaffold and contig N50 and total microsatellite content. Based on results from this analysis (described below), we chose to include all insect genomes regardless of sequencing platform or N50 statistics.

Preliminary inspection of our insect genomes suggested that some were highly incomplete (e.g., assembly size 2% of expected genome size). Because of this, we performed a second quality assessment comparing BUSCO (Benchmarking Universal Single-Copy Orthologs) scores and total microsatellite content in all insect genomes [27]. We used default settings for BUSCO in conjunction with the insect gene set. Scores were calculated as the proportion of genes searched that were found as complete genes (in either single or duplicate copies). These scores were then compared to the total microsatellite content in each genome to determine if there was a cutoff below which microsatellites were poorly inferred. Based on this approach, we reduced the number of genomes examined to 231.

*Phylogenetic data:* For downstream comparative analyses, we downloaded sequences for the four most frequently sequenced mitochondrial genes (12S, COI, COII, and cytochrome b) and four nuclear genes (18S, 28S, elongation factor 1, and arginine kinase). This yielded a dataset of 221 operational taxonomic units (OTUs) representing members of 12 of the 24 insect orders. All sequences were downloaded from GenBank (accession numbers in Table S2 (Supplementary Materials)). The sequences were aligned in MAFFT v.7 using default settings [28]. We used Gblocks 0.91b to remove ambiguously aligned sites from 12S, 18S, and 28S alignments, using options for less stringent selection, including allowing smaller final blocks, gap positions within blocks, and less strict flanking requirements [29]. This resulted in alignments for 12S, 18S, and 28S of 346 bp, 1442 bp, and 253 bp in length, respectively. We used MEGA to manually adjust the alignments of protein coding genes (COI, COII, elongation factor 1, cytochrome b, and arginine kinase) to ensure that the reading frame was maintained [30]. These alignments were 1463 bp, 683 bp, 1064 bp, 409 bp, and 1019 bp in length, respectively. For tree inferences in RAxML, alignments were concatenated (total length 6686 bp), while each gene was kept separate for Bayesian tree inference.

Rogue taxa, or taxa which are placed inconsistently with equal probability during phylogenetic inference due to insufficient or erroneous data, will often lead to overestimation of rates of trail evolution, similar to what is seen in supertrees [31,32]. To avoid this problem, we inferred 100 rapid bootstrap trees using RAxML-HPC v.8 on XSEDE using the CIPRES Science Gateway [33,34]. Using these trees, we calculated taxon instability index with Mesquite v 3.6 [35]. A high index value indicates that a taxon has variable placement among trees. By visual inspection of this distribution, we found that 92% of taxa have indices less than 5000; however, above this, instability quickly increases (Figure S1 (Supplementary Materials)). To ensure that our estimate of rates was conservative, we removed the 18 taxa with scores higher than this cutoff. Filtering our dataset based on BUSCO scores (discussed below), gene sequence availability, and taxonomic instability index scores led to a final dataset of 201 taxa for our Bayesian analysis.

We used BEAST v2.5.2 for the inference of time-calibrated phylogenies [36]. For a starting tree, we selected the best maximum likelihood tree from RAxML, which we converted to an ultrametric tree using nonparametric rate smoothing implemented in the function chronos in the R package APE [37]. We assumed a relaxed log-normal clock, a GTR substitution model with among-site rate variation modeled with a γ distribution, and a birth–death branching model. We estimated nucleotide substitution model parameters independently across four partitions: protein codon positions 1, 2, and 3, and the ribosomal positions. To calibrate divergence time estimation, we placed eight priors on node ages in the tree. Normal distributions with means and standard deviations were chosen to represent previous estimates of the ages of the root of the tree and the origin of Lepidoptera, Diptera, Hymenoptera, Coleoptera, Blattodea/Phasmatodea, Hemiptera, and Ephemeroptera/Odonata (Table S3 (Supplementary Materials)) [38]. Two independent BEAST runs were completed.

*Microsatellite and other trait data:* We used micRocounter v.1.1.0 to characterize the microsatellite content within the insect genome assemblies [39]. We recorded the number of dinucleotide (2mer), trinucleotide (3mer), tetranucleotide (4mer), pentanucleotide (5mer), and hexanucleotide (6mer) repetitive sequences. Default micRocounter settings for all parameters were used (2mers required six repeats, 3mers required four repeats, and 4–6mers required three repeats). We used a publicly available dataset to gather centromere type (holocentric or monocentric) and chromosome number for as many species as possible in our study [40,41]. We additionally gathered available genome size estimates from the Animal Genome Size Database [42].

*Estimating rates of microsatellite evolution:* We fit Brownian motion models to estimate rates of microsatellite evolution at several levels. All rate estimates described were generated using the restricted maximum likelihood approach using the ace function in the R package APE [37]. This function takes observed microsatellite content and the phylogeny, and it returns an ancestral state estimate for every node in the tree and the maximum likelihood estimate for the rate of evolution. For comparison, we fit the same model using the fitContinuous function in the R package Geiger v2.0.6.4 [43]. Rate estimates between these two approaches were qualitatively identical.

Firstly, we fit a model where we assumed a single rate of microsatellite evolution across the entire phylogeny. Next, we estimated rates individually for each order that had at least 10 species in our dataset (for both of these analyses, we fit our model based on microsatellite bp per Mbp). Finally, we calculated tip rates. Using the ancestral state estimates from our combined analysis of all data, tip rates were estimated by taking the difference in microsatellite content of a species and the ancestral state estimate for the node from which it descends. This value represents the change since the last speciation event sampled on our phylogeny (this was calculated based on the total bp of microsatellites estimated for each tip in the tree). This value was then divided by the branch length since that speciation event, providing an estimate for the recent rate of evolution in a species lineage.

*The impact of centromere type on microsatellite content and evolution:* We firstly tested whether species with holocentric and monocentric chromosomes have significantly different microsatellite content. We analyzed the quantity of each microsatellite size class (2–6mer), total microsatellite content, and microsatellite content per Mbp using a phylogenetic ANOVA implemented in Geiger [43]. The phylogenetic ANOVA was repeated for each tree from the posterior distribution. To calculate *p*-values, the observed F-statistic was compared to a null distribution generated from 100 simulations.

In addition to differences in microsatellite content, type of centromere may also affect the rate at which microsatellite content evolves. We tested for a difference in the rate of microsatellite evolution in species with holocentric and monocentric chromosomes using a censored rate test implemented in the brownieREML function in phytools v0.6-99 [44]. This allowed us to compare models where the continuous trait (microsatellite content) evolves at a single rate on all branches to a model where each state has independent rates of evolution (O’Meara et al. 2006). We used the function make.simmap in phytools to generate the stochastic maps (holocentric vs. monocentric states) that are used in brownieREML [44]. In construction of the stochastic map, we used a Markov model and allowed rates of transition between holocentric and monocentric to differ. To account for uncertainty in ancestral states, we repeated our analysis across 100 stochastic maps.

*Comparing rates and content to chromosome number and genome size:* We hypothesized that, if microsatellites are common in structural elements of chromosomes, then those species with more chromosomes would be expected to have greater microsatellite content. We analyzed the data using a phylogenetic linear model where microsatellite content in bp of microsatellite content/Mbp of genome was the response variable and chromosome number was the predictor variable. We also fit a phylogenetic linear model where the tip rate as described above was the response variable and chromosome number was the predictor variable. Both of these models were fit using the function phylolm in the R package phylolm v2.6 and used all 100 posterior distribution trees with 100 bootstraps for each tree [45].

We also assessed genome size as a predictor of microsatellite evolution. For this analysis, we used genome size in Mbp. We analyzed the data using a phylogenetic linear model where microsatellite content in Mbp was the response variable and genome size was the predictor variable. This analysis used the 100 posterior distribution trees with 100 bootstraps for each tree. We also fit a phylogenetic linear model where the tip rate as described above was the response variable and genome size was the predictor variable. Again, this analysis used the 100 posterior distribution trees with 100 bootstraps for each tree. Both of these models were fit using the function phylolm in the R package phylolm. [45]. All analyses were completed in R version 3.6.3 [46]. All tests were considered significant at α = 0.05. All data and code necessary for our analyses are available on Dryad (available upon acceptance).

## 3. Results

### 3.1. Data Quality and Collection

#### 3.1.1. Assembly Quality

Firstly, repetitive sequences were reanalyzed from a prior study with 71 vertebrates to determine the effect of sequencing platform (short, Sanger, and long) on microsatellite inference. We hypothesized that shorter sequencing may not be able to fully span repetitive regions, generating a pattern of estimates of lower microsatellite content in genomes assembled from short-read sequencing platforms. However, we found no significant difference in microsatellite content among genomes assembled with these three classes of platforms (Figure S2 (Supplementary Materials)). We also investigated the impact of N50 for both contigs and scaffolds on estimated microsatellite content. Using a linear model, we found no significant effect of either of these measures on microsatellite content (*p*-value for N50 contig = 0.70 and *p*-value for N50 scaffold = 0.18). Finally, we fit a linear model where microsatellite content was the response variable and genome size was the predictor variable and found no significance between them (*p*-value = 0.24; Figure S3 (Supplementary Materials)). These results suggest no strong biases in estimates of microsatellite content among these vertebrate genomes. While this suggests that it may be acceptable to use existing genome assemblies for comparative analyses, we feared that some of the 304 insect genomes we downloaded may be more poorly assembled than the vertebrate genomes we examined. For this reason, we investigated the relationship between BUSCO scores and estimated microsatellites content. We found an unexpected pattern where some genomes with very low BUSCO scores (e.g., less than 0.05) had the highest microsatellite content (Figure S4 (Supplementary Materials)). These low-scoring genome assemblies also exhibited both some of the smallest and some of the largest genome assemblies in our collection of genomes. Genomes with BUSCO scores greater than 0.1 did not appear to have any consistent bias in microsatellite content; however, we chose to conservatively retain only those genomes with BUSCO scores of at least 0.90, reasoning that these genomes were most likely the most well assembled in our dataset. Using this threshold, we discarded 83 genomes, and all downstream analyses were performed on the remaining 221 genomes. As mentioned above, this was further reduced to 201 for all analyses involving our phylogeny due to elevated taxonomic instability scores during tree inference.

#### 3.1.2. Phylogenetic Reconstruction

Both MCMC chains converged on a parameter space with equal likelihood by 100 million generations. The chains were then allowed to run for an additional 100 million generations to ensure that neither chain discovered an area of higher likelihood. Convergence was evaluated using Tracer v1.7.1 [47]. The first 75% of each MCMC was discarded as burn-in, and 50 trees were randomly sampled from the post-burn-in portion of each MCMC. These 100 trees represent our posterior distribution of trees and were used in all downstream analyses. The phylogenies inferred were consistent with a comprehensive order-level phylogeny [38].

#### 3.1.3. Microsatellite Content and Rates

**Figure 1:**
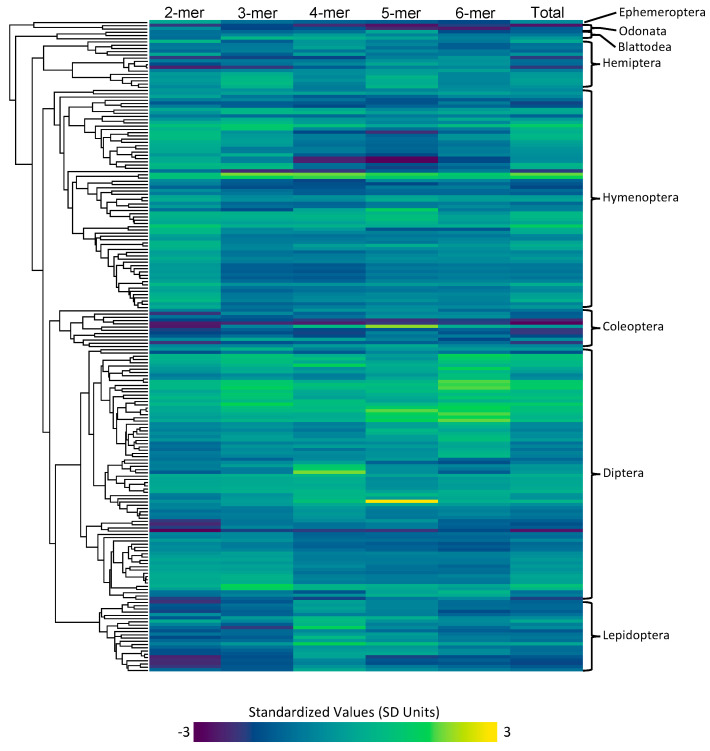
The inferred microsatellite counts for each type (2mer, 3mer, 4mer, 5mer, 6mer, and total), with cool colors representing lower microsatellite content and warm colors representing higher microsatellite content. The values are standardized across all types. The phylogeny depicting the row that corresponds to each species can be seen on the left, and the orders that each of these species encompass is on the right.

Microsatellite content for each type of microsatellite (2mer, 3mer, 4mer, 5mer, 6mer) was measured for each of the species in our dataset (Figure 1). The highest total microsatellite content was found in *Blatella germanica* with a total of 19.03 Mbp of microsatellite content. The lowest microsatellite content was found in *Belgica antarctica* with a total of 0.17 Mbp of microsatellite content. Across all insects, we found that 2mers are the most abundant type of microsatellite, accounting for an average of 1.06 Mbp of the average insect genome assembly.

**Figure 2:**
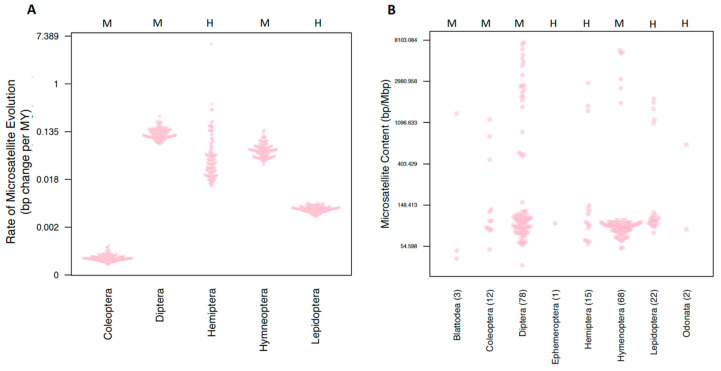
Comparing microsatellite content and rates of evolution among orders. Both *y*-axes are measured in a log scale. The centromere type present in an order is indicated with an H or M at the top of the plot for holocentric and monocentric, respectively. Orders are indicated on the horizontal axis. (**A**) The rate of microsatellite evolution for all orders with at least 10 representatives. For each order, 100 estimates derived from each of the 100 trees is plotted. (**B**) Microsatellite content for all species included in comparative analyses.

Our estimates of order rates revealed striking variation in rates of evolution (Figure 2A). Coleoptera exhibited the lowest rates of microsatellite evolution (σ^2^ = 0.006 × 10^5^), while Diptera and Hemiptera exhibited the highest rates of microsatellite evolution (σ^2^ = 1.278 × 10^5^ and 1.157 × 10^5^, respectively). Next, we estimated tip rates of microsatellite evolution. Tip rates (in units of bp change per million years) for most species were normally distributed around zero. However, two hemipterans, *Pseudococcus longispinus* and *Paracoccus marginatus,* both exhibited strikingly negative tip rate values (−3.3 × 10^−6^ and −3.7 × 10^−6^ respectively). While, alone, these values may not seem striking, these numbers are 45 and 51 times larger, respectively, than the relative mean tip rate observed in our dataset. These two species also exhibited a considerably smaller genome size than is typical for hemipterans (average = 490 Mbp) with a genome size of 285 Mbp for *P. longispinus* and 191 Mbp for *P. marginatus*.

### 3.2. Comparative Analyses

#### 3.2.1. Order

We firstly tested whether different orders of insects had different microsatellite content using both a standard and a phylogenetically corrected ANOVA. The response variables were the raw count of bp of each microsatellite type (2–6mer), the total sum of all microsatellites, and the proportion of the genome that is composed of microsatellite content. This proportion was calculated by taking the total raw microsatellite content divided by the assembly size. While we found that standard ANOVAs returned significant results for six of the seven response variables, none of these were significant after correction for phylogenetic history (Table S4 (Supplementary Materials)). This result was not unexpected upon examining the distribution of microsatellite proportions (Figure 2B).

#### 3.2.2. Centromere Type

**Figure 3:**
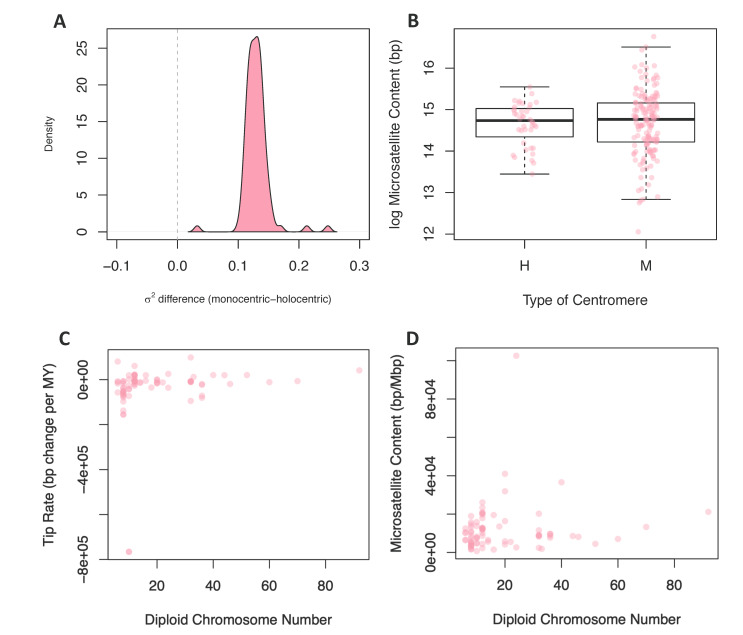
Comparing rates and content to centromere type and chromosome number. (**A**) The difference between the monocentric and holocentric rate predicted under a Brownian motion model for the 100 posterior distribution trees. (**B**) The difference between the microsatellite content in base pairs between holocentric and monocentric species. The *y*-axis is in the log scale. (**C**) The relationship between microsatellite evolution rates and the diploid chromosome number for each species. (**D**) The relationship between the microsatellite content in bp/Mbp and the diploid chromosome number for each of the species.

We reasoned that, due to the repetitive nature of centromeric sequences, species with different centromere types may have distinct tempos and modes of evolution. Using stochastic maps of centromere type evolution, we performed a censored rate test to determine if rates of microsatellite evolution were significantly different in lineages with these two types of centromeres. Out of the 100 posterior distribution trees, 99 favored a two-rate model. The rate estimates for microsatellite evolution were higher in lineages with monocentric chromosomes for all trees, including the one tree that did not support a two-rate model as significantly better (Figure 3A). We also compared the mean microsatellite content (2–6mer and total content) of lineages with monocentric and holocentric chromosomes using a standard and phylogenetically corrected ANOVA (Figure 3B). In no cases were monocentric and holocentric lineages significantly different (Table S5 (Supplementary Materials)).

#### 3.2.3. Chromosome Number and Genome Size

**Figure 4:**
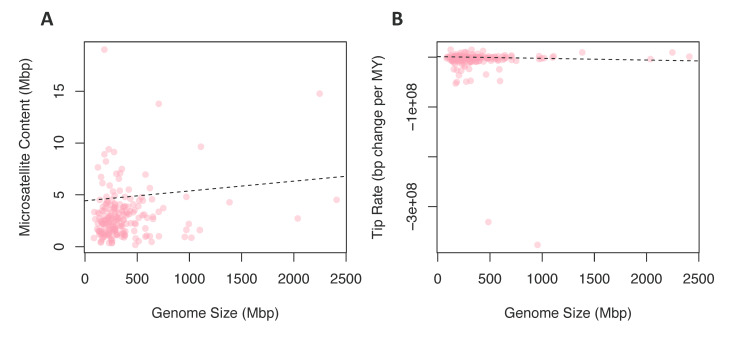
Comparing rates and content to genome size. (**A**) The relationship between microsatellite content in Mbp and the genome size in Mbp for each species. (**B**) The relationship between microsatellite evolution rates and the genome size in Mbp for each species.

To test for a contingency between these traits, we fit linear models where chromosome number or genome size were predictor variables and tip rates of microsatellite evolution or microsatellite content were response variables; in all cases, linear models were fit while correcting for phylogeny. We found no significant relationship between chromosome number and rates of microsatellite evolution (Figure 3C) or microsatellite content (Figure 3D). In contrast, when we tested genome size as the predictor variable for microsatellite content, 96 of 100 phylogenies produced a significant result (Figure 4A). For 99 of the models, the slope of this relationship was positive, indicating that increased genome size is associated with increased microsatellite content. Likewise, when the response variable was rate of microsatellite evolution, 53 out of 100 trees produced a significant result (Figure 4B). However, in this case, the increased genome size was associated with a very small decrease in the rate of microsatellite evolution in 84 of the 100 trees.

## 4. Discussion

While most other studies focused on microsatellite content and rates of evolution across specific taxa, we investigated several possible predictors that may account for different microsatellite dynamics among orders of insects. We investigated the impact of chromosome number, centromere type, and genome size on microsatellite content and rates of evolution. We found significant differences in the microsatellite content among taxa, even those closely related. We also found that, across all sequenced genomes, microsatellite content scales with genome size. Finally, we showed that species with monocentric chromosomes have significantly higher rates of microsatellite evolution.

*The impacts of genome assembly quality:* One of our initial questions in using genome assemblies to understand microsatellite evolution was the potential impact of assembly quality on downstream analyses. We addressed this question using two datasets. Firstly, we analyzed data from a previous microsatellite study using vertebrate genomes [26]. Then, we evaluated the inference of microsatellites across all sequenced insect genomes. Our results demonstrated that, for the vertebrate data, microsatellite content inference is not biased by any quality metric we analyzed. In contrast, for insect genomes, we found that assemblies with very low BUSCO scores exhibited an exceedingly wide range of microsatellites. This pattern could be a result of some genomes with exceptionally large amounts of repetitive elements being both difficult to assemble and greatly enriched for microsatellites. However, the fact that genomes with these low BUSCO scores exhibit both higher and lower microsatellite content than is typical of well-assembled genomes suggests that this may be an artefact of a poor assembly. Furthermore, these species do not all have larger genomes than is typical for their clades, which would be expected if the pattern was driven by a massive expansion of repetitive elements. Based on this logic, we excluded genomes with low BUSCO scores and suggest that future studies with any genome used for investigations of microsatellite content should have a BUSCO score in excess of 90 to ensure that the results reflect biological reality rather than the poor quality of the assembly.

### 4.1. Traits

*Centromere type*: Theoretical work showed that microsatellites should expand in regions such as the centromere where there is suppression of recombination and weak selective pressure on the array length [48]. Two primary types of centromeres are present across the tree of life and within insects [40,49]: holocentric chromosomes where centromere function is diffuse across the entire length of the chromosome and monocentric chromosomes where a single region of the chromosome functions as the centromere. These two types of centromeres lead to fundamentally different behavior with regard to resolution of chiasma that form during recombination. While monocentric chromosomes can segregate with multiple chiasma even in a single arm, holocentric chromosomes with more than one or two chiasma per chromosome fail to segregate properly [50]. Furthermore, centromeric regions in both types of chromosomes exhibit reduced recombination [51]. This difference could lead to more regions of low recombination in holocentric species and greater opportunity for expansion of microsatellites relative to monocentric species. This might suggest that holocentric species would have larger genomes due to the proliferation of microsatellites and other repetitive sequences. However, analyses across a range of taxa do not support a difference in genome size between holo- and monocentric species [52]. Another potential cause of differences in monocentric and holocentric species could be differences in DNA replication mechanics. Some evidence suggests that the distribution of translation initiation sites may vary based on centromere type [53].

In some plants and nematodes, studies suggested a higher satellite content in species with holocentric chromosomes [53,54]. However, the most taxonomically broad analysis of satellite content comparing holocentric and monocentric species supports lower satellite content in holocentric species [55]. These studies focused on all satellite content, and the results were largely driven by mini- or macrosatellites. Our results suggest that, within insects, there is no significant difference in microsatellite content when comparing holocentric and monocentric species. This suggests that microsatellites do not play a central role in defining centromeric regions and that the selective forces constraining microsatellite expansion may be similar regardless of the centromere type.

Based on the similarity in microsatellite content that we observed among holocentric and monocentric lineages, we hypothesized that they would also demonstrate similar rates of evolution. However, when we fit a Brownian motion model of evolution to microsatellite content, we inferred consistently higher rates in monocentric species than in holocentric species. Examining the total microsatellite content of all species in our study, we can see that the monocentric orders Hymenoptera and Diptera both exhibit a range of microsatellite content that exceeds all holocentric orders combined (Figure 2B). We suggest that these orders likely drive much of the signal for higher rates of microsatellite evolution in monocentric species. However, not all monocentric clades exhibit high rates of microsatellite evolution; Coleoptera actually exhibits the lowest rate of microsatellite evolution of any order that we studied (Figure 2A). This highlights one of the dangers of comparative approaches that test for differences in rates of continuous trait evolution in two states of a discrete trait, i.e., a small portion of a phylogeny may contain such a strong signal that any binary trait mapped onto that portion of the phylogeny will be positively correlated with high rates. This is similar to the source of inflated rates of false positives that recently caused upheaval in attempts to detect differential diversification under BiSSE models [56]. More broadly, the magnitude of variation we observed is striking and was not fully explained by any of the explanatory variables that we examined. This suggests that additional factors must play an important role in determining the observed differences among species.

*Chromosome number*: Based on detailed analyses of Diptera genomes, microsatellites appear to be rare in heterochromatic portions of the genome [57,58]. However, heterochromatic regions are generally enriched for a variety of repetitive sequences [59,60]. Centromeres and telomeres, as well as the regions adjacent to them, are often heterochromatic, and the number of these structural regions scales with the number of chromosomes a genome contains. We might also expect chromosome number to correlate with rate of evolution where more recombination occurs in species with many chromosomes. With each recombination event, there is an opportunity for misalignment of repeat units, which would lead to a longer or shorter locus in the resulting gametes. In our analysis, we found no significant correlation between chromosome number and either microsatellite content or microsatellite rate of evolution. We interpret this as evidence that these regions are not a “hot spot” for microsatellite accumulation in most insect species, and that changes in microsatellite length due to recombination errors are rare. However, we note that centromeric and telomeric regions are difficult to assemble regions of the genome and may become more difficult to assemble as the number of chromosomes increases. As such, the use of whole genome assemblies rather than raw reads may reduce our ability to detect a concentration of microsatellites in these regions. More broadly, the results that we presented are likely most applicable to the tempo and mode of microsatellite evolution in euchromatic portions of the genome.

*Genome size*: There is large variation in genome size among eukaryotes; even closely related species often have strikingly different genome sizes [61]. This variation in eukaryotes is not predictive of complexity, ploidy level, or the number of protein-coding genes [62,63]. The evolution of genome size can be directly affected by a variety of processes, e.g., insertions, deletions, polysomy, proliferation of transposons (reviewed in Reference [64]). A correlation between microsatellite content and genome size may be produced in two distinctly different fashions. Firstly, microsatellite expansion or contraction may lead directly to changes in genome size, or broader deletion or insertion processes may drive a global change in genome size that impacts microsatellites as a byproduct. The relationship between microsatellite content and genome size was confirmed in many species [13,65,66]. Although our study could not distinguish among the possible drivers of this correlation, we did find a signal for contingency among genome size and microsatellite content (Figure 4A). Furthermore, we found a correlation between the rate of microsatellite evolution and genome size. We interpret this as support for the proportional model of genome size evolution [67]. This hypothesis suggests that variation in rates of genome size evolution (and, in turn, genome size) is driven by broad rate differences that are a function of the genome size, such that species with large genomes also have higher rates of genome size evolution.

### 4.2. Clades

*Content*: Our analysis demonstrated that microsatellite content is highly variable across species (Figure 1). However, differences among orders were not statistically significant once we corrected for the phylogenetic history. Instead, we found a pattern where often even closely related species exhibit striking differences in microsatellite content. For instance, within the fly family Tephritidae, *Ceratitis capitata* has 10 times more 5mer repeats than the four *Bactrocera* species. *C. capitata* even has three times more 5mer repeats than *Rhagoletis zephyria* despite the *Rhagoletis* species having 30% more total microsatellite content. This pattern suggests that microsatellite content can evolve rapidly and confirms previous studies that suggested that species often have unique microsatellite landscapes [68].

*Rates*: Although we found that clades with monocentric chromosomes had higher rates of microsatellite evolution, they also exhibited striking variation in rates of evolution. In fact, among all orders evaluated, the monocentric orders Diptera and Coleoptera had the highest and lowest rate of microsatellite evolution, respectively (Figure S5 (Supplementary Materials)). In contrast, both holocentric orders had intermediate rates. The broad range of rates estimated within monocentric clades suggests that monocentricity is unlikely to be the driving force responsible for rate differences among clades. Instead, we suggest that there must be other traits that are present in some monocentric clades that contribute to a higher rate estimate in lineages with this type of chromosome.

## 5. Conclusions

The molecular mechanism via which microsatellite content evolves is well-understood; however, the drivers of variation in patterns of microsatellite evolution across large clades is not. This study is, to our knowledge, the first to test how centromere type, chromosome number, and genome size impact clade-level microsatellite content and rates of evolution. Our results show that there is large variation both in microsatellite content and in type of microsatellite repeats within and among orders. Furthermore, the rates at which this microsatellite content evolves differ among orders and centromere type. Based on our study, we suggest that Coleoptera and Diptera are particularly good clades to compare as they exhibit the largest difference in rates of evolution. The advent of long-read sequencing technology coupled with approaches that provide genome wide scaffolding will lead to a vast increase in the number and completeness of genomes that are publicly available. These new assemblies will have the potential to evaluate the evolution of microsatellites across the entire genome rather than being concentrated in euchromatic portions, as is currently the case. Approaches like those we used that allow for an evolving trait to impact the rate of evolution of a second trait could leverage these genomes to reveal the impact of a broad range of characters (e.g., TEs, structural elements, codon bias, recombination rates) on the evolution of microsatellite content.

## Supplementary Materials

The following are available online at https://www.mdpi.com/2073-4425/11/8/945/s1: Figure S1. Taxonomic instability indices; Figure S2. Microsatellite inference and the type of sequencing; Figure S3. Measures of the quality of genomes; Figure S4. BUSCO scores; Figure S5. Tip rates; Table S1. Genome accession numbers; Table S2. Gene accession numbers; Table S3. Node constraints; Table S4. ANOVA results of microsatellite content by order; Table S5. ANOVA results of microsatellite content by centromere type.

## References

[1] Metzgar D., Bytof J., Wills C. (2000). Selection against frameshift mutations limits microsatellite expansion in coding DNA. Genome Res..

[2] Edwards Y.J.K., Elgar G., Clark M.S., Bishop M.J. (1998). The identification and characterization of microsatellites in the compact genome of the japanese pufferfish, Fugu rubripes: Perspectives in functional and comparative genomic analyses. J. Mol. Biol..

[3] Moore H., Greenwell P.W., Liu C.-P., Arnheim N., Petes T.D. (1999). Triplet repeats form secondary structures that escape DNA repair in yeast. Proc. Natl. Acad. Sci. USA.

[4] Dokholyan N.V., Buldyrev S.V., Havlin S., Stanley H.E. (2000). Distributions of dimeric tandem repeats in non-coding and coding DNA sequences. J. Theor. Biol..

[5] Zhang L., Zuo K., Zhang F., Cao Y., Wang J., Zhang Y., Sun X., Tang K. (2006). Conservation of noncoding microsatellites in plants: Implication for gene regulation. BMC Genom..

[6] Fujimori S., Washio T., Higo K., Ohtomo Y., Murakami K., Matsubara K., Kawai J., Carninci P., Hayashizaki Y., Kikuchi S. (2003). A novel feature of microsatellites in plants: A distribution gradient along the direction of transcription. FEBS Lett..

[7] Ellegren H. (2000). Microsatellite mutations in the germline: Implications for evolutionary inference. Trends Genet..

[8] Slatkin M. (1995). A measure of population subdivision based on microsatellite allele frequencies. Genetics.

[9] Criscione C.D., Vilas R., Paniagua E., Blouin M.S. (2011). More than meets the eye: Detecting cryptic microgeographic population structure in a parasite with a complex life cycle. Mol. Ecol..

[10] Ballantyne K.N., Goedbloed M., Fang R., Schaap O., Lao O., Wollstein A., Choi Y., van Duijn K., Vermeulen M., Brauer S. (2010). Mutability of Y-chromosomal microsatellites: Rates, characteristics, molecular bases, and forensic implications. Am. J. Hum. Genet..

[11] Blouin M.S. (2003). DNA-based methods for pedigree reconstruction and kinship analysis in natural populations. Trends Ecol. Evol..

[12] Highnam G., Franck C., Martin A., Stephens C., Puthige A., Mittelman D. (2012). Accurate human microsatellite genotypes from high-throughput resequencing data using informed error profiles. Nucleic Acids Res..

[13] Field D., Wills C. (1998). Abundant microsatellite polymorphism in Saccharomyces cerevisiae, and the different distributions of microsatellites in eight prokaryotes and S. cerevisiae, result from strong mutation pressures and a variety of selective forces. Proc. Natl. Acad. Sci. USA.

[14] Pearson C.E., Sinden R.R. (1996). Alternative structures in duplex DNA formed within the trinucleotide repeats of the myotonic dystrophy and fragile X loci. Biochemistry.

[15] Schmidt T., Heslop-Harrison J.S. (1996). The physical and genomic organization of microsatellites in sugar beet. Proc. Natl. Acad. Sci. USA.

[16] Hoffman E.K., Trusko S.P., Murphy M., George D.L. (1990). An S1 nuclease-sensitive homopurine/homopyrimidine domain in the c-Ki-ras promoter interacts with a nuclear factor. Proc. Natl. Acad. Sci. USA.

[17] Chamberlain N.L., Driver E.D., Miesfeld R.L. (1994). The length and location of CAG trinucleotide repeats in the androgen receptor N-terminal domain affect transactivation function. Nucleic Acids Res..

[18] Lue N.F., Buchman A.R., Kornberg R.D. (1989). Activation of yeast RNA polymerase II transcription by a thymidine-rich upstream element in vitro. Proc. Natl. Acad. Sci. USA.

[19] Sandberg G., Schalling M. (1997). Effect of in vitro promoter methylation and CGG repeat expansion on FMR-1 expression. Nucleic Acids Res..

[20] Rubinsztein D.C., Leggo J., Coetzee G.A., Irvine R.A., Buckley M., Ferguson-Smith M.A. (1995). Sequence variation and size ranges of CAG repeats in the Machado-Joseph disease, spinocerebellar ataxia type 1 and androgen receptor genes. Hum. Mol. Genet..

[21] Eisen J., Goldstein D., Schlotterer C. (1999). Mechanistic basis for microsatellite instability. Microsatellites: Evolution and Applications.

[22] Klintschar M., Dauber E.M., Ricci U., Cerri N., Immel U.D., Kleiber M., Mayr W.R. (2004). Haplotype studies support slippage as the mechanism of germline mutations in short tandem repeats. Electrophoresis.

[23] Tóth G., Gáspári Z., Jurka J. (2000). Microsatellites in different eukaryotic genomes: Survey and analysis. Genome Res..

[24] Bell C.J., Ecker J.R. (1994). Assignment of 30 microsatellite loci to the linkage map of Arabidopsis. Genomics.

[25] Neff B.D., Gross M.R. (2001). Microsatellite evolution in vertebrates: Inference from AC dinucleotide repeats. Evolution.

[26] Adams R.H., Blackmon H., Reyes-Velasco J., Schield D.R., Card D.C., Andrew A., Castoe T.A. (2016). Microsatellite landscape evolutionary dynamics across 450 million years of vertebrate genome evolution. Genome.

[27] Simão F.A., Waterhouse R.M., Ioannidis P., Kriventseva E.V., Zdobnov E.M. (2015). BUSCO: Assessing genome assembly and annotation completeness with single-copy orthologs. Bioinformatics.

[28] Katoh K., Rozewicki J., Yamada K.D. (2019). MAFFT online service: Multiple sequence alignment, interactive sequence choice and visualization. Brief. Bioinform..

[29] Talavera G., Castresana J. (2007). Improvement of phylogenies after removing divergent and ambiguously aligned blocks from protein sequence alignments. Syst. Biol..

[30] Tamura K., Peterson D., Peterson N., Stecher G., Nei M., Kumar S. (2011). MEGA5: Molecular evolutionary genetics analysis using maximum likelihood, evolutionary distance, and maximum parsimony methods. Mol. Biol. Evol..

[31] Thomson R.C., Shaffer H.B. (2010). Sparse supermatrices for phylogenetic inference: Taxonomy, alignment, rogue taxa, and the phylogeny of living turtles. Syst. Biol..

[32] Rabosky D.L. (2015). No substitute for real data: A cautionary note on the use of phylogenies from birth–death polytomy resolvers for downstream comparative analyses. Evolution.

[33] Miller M.A., Pfeiffer W., Schwartz T. Creating the CIPRES Science Gateway for inference of large phylogenetic trees. Proceedings of the 2010 Gateway Computing Environments Workshop (GCE).

[34] Stamatakis A. (2014). RAxML version 8: A tool for phylogenetic analysis and post-analysis of large phylogenies. Bioinformatics.

[35] Maddison W., Maddison D. (2011). Mesquite: A Modular System for Evolutionary Analysis. http://mesquiteproject.org.

[36] Drummond A.J., Rambaut A. (2007). BEAST: Bayesian evolutionary analysis by sampling trees. BMC Evol. Biol..

[37] Paradis E., Claude J., Strimmer K. (2004). APE: Analyses of phylogenetics and evolution in R language. Bioinformatics.

[38] Misof B., Liu S., Meusemann K., Peters R.S., Donath A., Mayer C., Frandsen P.B., Ware J., Flouri T., Beutel R.G. (2014). Phylogenomics resolves the timing and pattern of insect evolution. Science.

[39] Lo J., Jonika M.M., Blackmon H. (2019). Microcounter: Microsatellite characterization in genome assemblies. G3 Genes Genomes Genet..

[40] Blackmon H., Ross L., Bachtrog D. (2017). Sex determination, sex chromosomes, and karyotype evolution in insects. J. Hered..

[41] The Tree of Sex Consortium (2014). Tree of Sex: A database of sexual systems. Sci. Data.

[42] Gregory T.R. (2020). Animal Genome Size Database. http://www.genomesize.com.

[43] Harmon L.J., Weir J.T., Brock C.D., Glor R.E., Challenger W. (2008). GEIGER: Investigating evolutionary radiations. Bioinformatics.

[44] Revell L.J. (2012). Phytools: An R package for phylogenetic comparative biology (and other things). Methods Ecol. Evol..

[45] Ho L.S.T., Ane C., Lachlan R., Tarpinian K., Feldman R., Yu Q., van der Bijl W., Vos R H.M.L. Package ‘phylolm’. http://www.cran.r-project.org/web/packages/phylolm/index.html.

[46] Andy B., Mikko K. (2019). A Language and Environment for Statistical Computing.

[47] Rambaut A., Drummond A.J., Xie D., Baele G., Suchard M.A. (2018). Posterior summarization in bayesian phylogenetics using tracer 1.7. Syst. Biol..

[48] Stephan W. (1986). Recombination and the evolution of satellite DNA. Genet. Res..

[49] Melters D.P., Paliulis L.V., Korf I.F., Chan S.W. (2012). Holocentric chromosomes: Convergent evolution, meiotic adaptations, and genomic analysis. Chromosome Res..

[50] Nokkala S., Kuznetsova V., Maryanska-Nadachowska A., Nokkala C. (2004). Holocentric chromosomes in meiosis. I. Restriction of the number of chiasmata in bivalents. Chromosome Res..

[51] Cuacos M., Franklin H., Chris F., Heckmann S. (2015). Atypical centromeres in plants—What they can tell us. Front. Plant Sci..

[52] Mandrioli M., Carlo Manicardi G. (2012). Unlocking holocentric chromosomes: New perspectives from comparative and functional genomics?. Curr. Genom..

[53] Heckmann S., Macas J., Kumke K., Fuchs J., Schubert V., Ma L., Novak P., Neumann P., Taudien S., Platzer M. (2013). The holocentric species L uzula elegans shows interplay between centromere and large-scale genome organization. Plant J..

[54] Subirana J.A., Messeguer X. (2013). A satellite explosion in the genome of holocentric nematodes. PLoS ONE.

[55] Melters D.P., Bradnam K.R., Young H.A., Telis N., May M.R., Ruby J.G., Sebra R., Peluso P., Eid J., Rank D. (2013). Comparative analysis of tandem repeats from hundreds of species reveals unique insights into centromere evolution. Genome Biol..

[56] Rabosky D.L., Goldberg E.E. (2015). Model inadequacy and mistaken inferences of trait-dependent speciation. Syst. Biol..

[57] Bachtrog D., Weiss S., Zangerl B., Brem G., Schlötterer C. (1999). Distribution of dinucleotide microsatellites in the Drosophila melanogaster genome. Mol. Biol. Evol..

[58] Lowenhaupt K., Rich A., Pardue M. (1989). Nonrandom distribution of long mono-and dinucleotide repeats in Drosophila chromosomes: Correlations with dosage compensation, heterochromatin, and recombination. Mol. Cell. Biol..

[59] Charlesworth B., Sniegowski P., Stephan W. (1994). The evolutionary dynamics of repetitive DNA in eukaryotes. Nature.

[60] Yunis J.J., Yasmineh W.G. (1971). Heterochromatin, satellite DNA, and cell function. Science.

[61] Hartl D.L. (2000). Molecular melodies in high and low C. Nat. Rev. Genet..

[62] Mirsky A., Ris H. (1951). The desoxyribonucleic acid content of animal cells and its evolutionary significance. J. Gen. Physiol..

[63] Lynch M., Conery J.S. (2003). The origins of genome complexity. Science.

[64] Petrov D.A. (2001). Evolution of genome size: New approaches to an old problem. TRENDS Genet..

[65] Kubis S., Schmidt T., Heslop-Harrison J.S. (1998). Repetitive DNA elements as a major component of plant genomes. Ann. Bot..

[66] Primmer C.R., Raudsepp T., Chowdhary B.P., Møller A.P., Ellegren H. (1997). Low frequency of microsatellites in the avian genome. Genome Res..

[67] Oliver M.J., Petrov D., Ackerly D., Falkowski P., Schofield O.M. (2007). The mode and tempo of genome size evolution in eukaryotes. Genome Res..

[68] Rubinsztein D.C., Amos W., Leggo J., Goodburn S., Jain S., Li S.-H., Margolis R.L., Ross C.A., Ferguson-Smith M.A. (1995). Microsatellite evolution—Evidence for directionality and variation in rate between species. Nat. Genet..

