# Supplementary material for: Mode and Tempo of Microsatellite Evolution across 300 Million Years of Insect Evolution
Source: Genes (Basel). 2020 Aug 16;11(8):945. doi: 10.3390/genes11080945 (PMC7464534; doi:10.3390/genes11080945)
Supplement: Supplementary file 1 [file genes-11-00945-s001.pdf]

Supplement

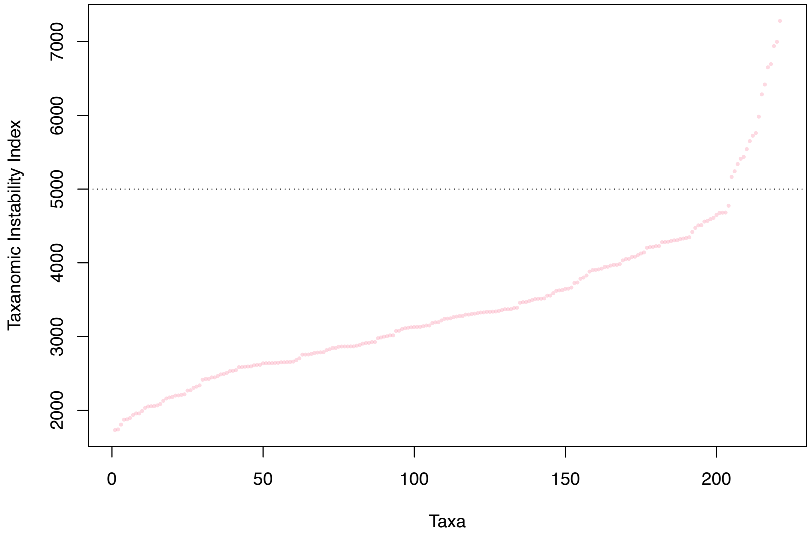

Supplemental Figure 1. Taxonomic instability indices based on 100 maximum likelihood tree inferences. The dashed line shows the chosen cutoff, an index of 5000. Most taxa, 92%, fall below this while above this value increases quickly.

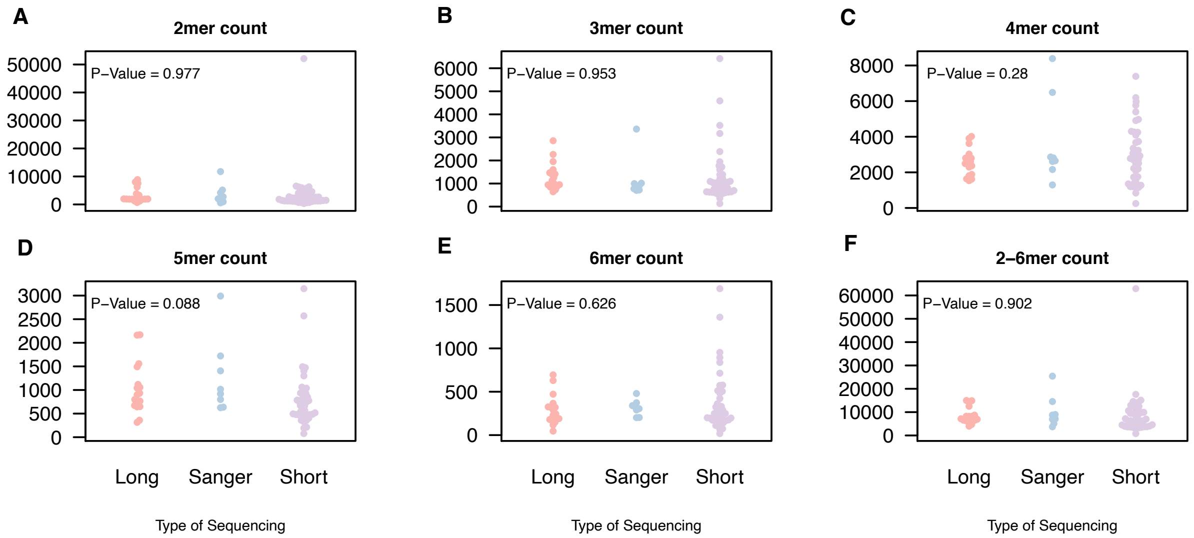

Supplemental Figure 2. Microsatellite inference and the type of sequencing (short, Sanger, long) using vertebrate genome dataset. This shows the relationship between inference of 2-mer, 3-mer, 4-mer, 5-mer, 6-mer, and total (2-6mer) microsatellite count and the types of sequencing with phylogenetic ANOVA values in the upper left hand for each type.

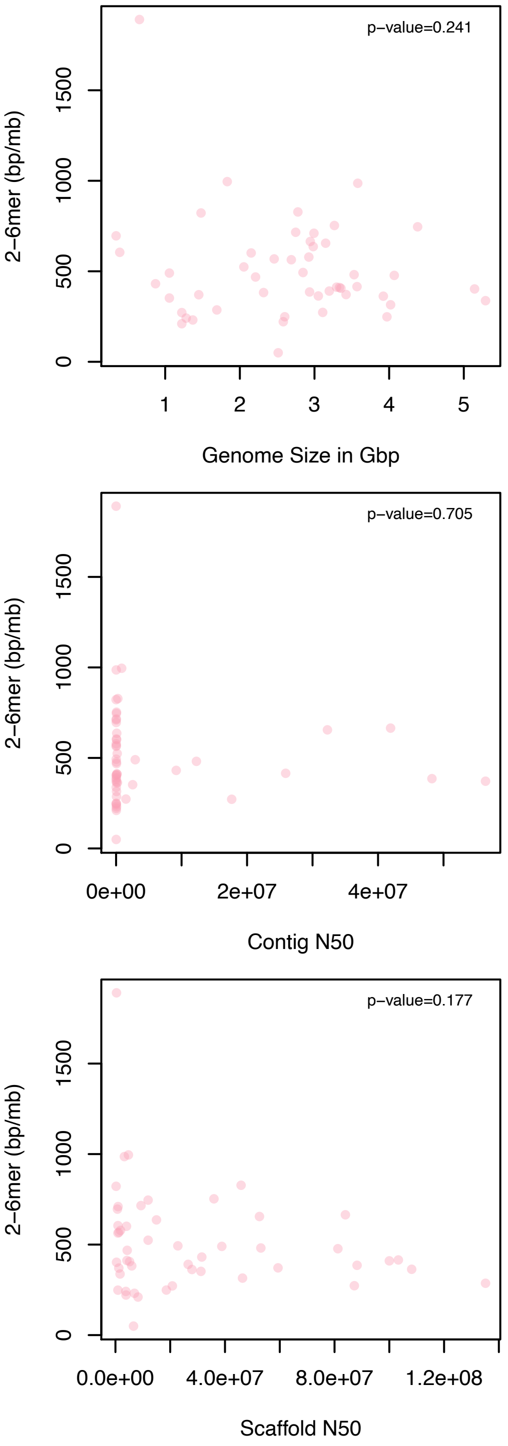

Supplemental Figure 3. Measures of the quality of genomes (scaffold N50, contig N50, and genome size) and microsatellite inference. Linear model p-values are indicated in the upper right-hand corner for each of the analyses.

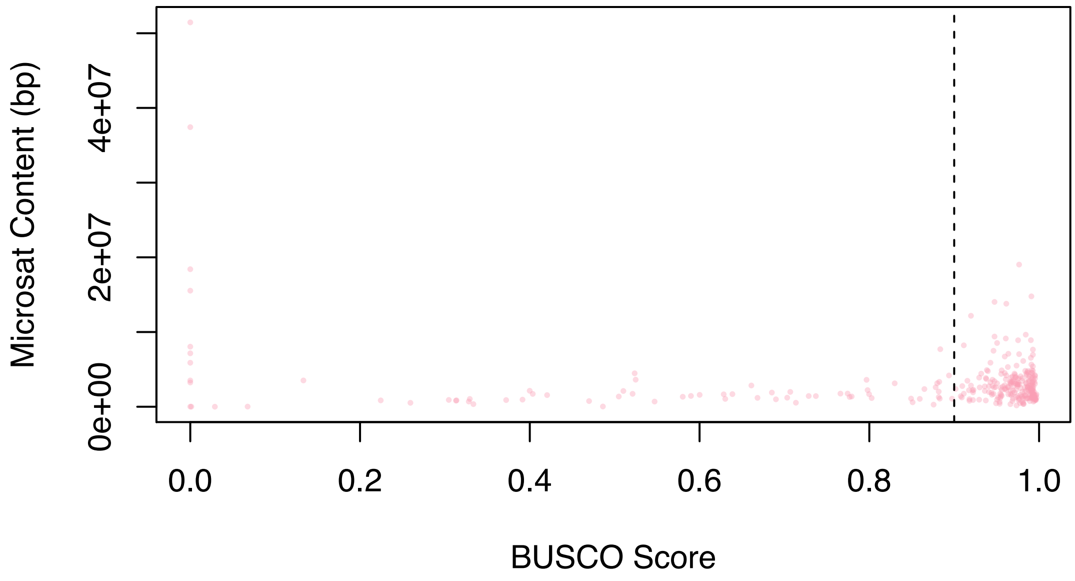

Supplemental Figure 4. BUSCO scores and microsatellite content (bp/Mbp). The vertical black line indicates the threshold of 90 that was required for inclusion in our study. There were 83 genomes that were discarded based on this threshold.

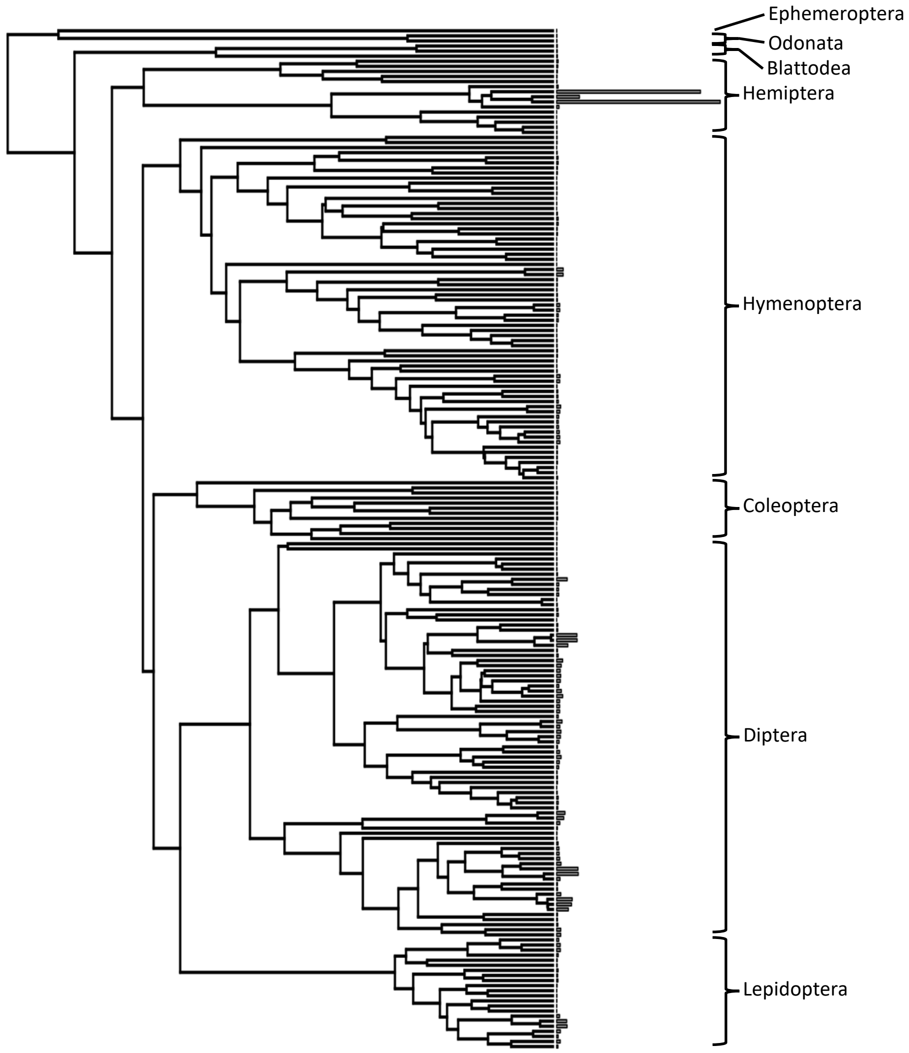

Supplemental Figure 5. Tip rates for microsatellite evolution across each of the taxa in our phylogeny. The bars at the end of each tip represents the absolute value of the microsatellite evolution tip rates for each taxa.

Supplemental Table 1. All genomes and accession numbers for the 303 insect species that are included in the analyses. Genome assemblies were downloaded from NCBI (https://www.ncbi.nlm.nih.gov), ENSEMBL (https://useast.ensembl.org/index.html), and Baylor HGC (https://www.hgsc.bcm.edu/arthropods/i5k).

Order	Species	Accession	Notes
Lepidoptera	*Achalarus lyciades*	MOOZ01000001.1	
Hymenoptera	*Acromyrmex echinatior*	NW_011623521.1	
Hemiptera	*Acyrthosiphon pisum*	GL349630	
Diptera	*Aedes aegypti*	GCF_002204515.2_AaegL5.0	
Diptera	*Aedes albopictus*	NW_017855997.1	
Coleoptera	*Aethina tumida*	NW_017852934	
Coleoptera	*Agrilus planipennis*	NW_017259594	
Coleoptera	*Aleochara bilineata*	NBZA01000001	
Plecoptera	*Amphinemura sulcicollis*	LVVV01000001	
Lepidoptera	*Amyelois transitella*	NW_013534156	
Diptera	*Anopheles alnimanus*	CM008152	
Diptera	*Anopheles aquasalis*	NJHH01000001	
Diptera	*Anopheles arabiensis*	KB704125	
Diptera	*Anopheles atroparvus*	KI421882	
Diptera	*Anopheles christyi*	KB673756	
Diptera	*Anopheles coluzzii*	EQ097730	
Diptera	*Anopheles cracens*	KZ067108	
Diptera	*Anopheles culicifacies*	KI422464	
Diptera	*Anopheles darlingi*	ADMH00000000	
Diptera	*Anopheles dirus*	KB672490	
Diptera	*Anopheles epiroticus*	KB669613	
Diptera	*Anopheles farauti*	KI915040	
Diptera	*Anopheles funestus*	KB668221	

Order	Species	Accession	Notes
Diptera	*Anopheles gambiae*	AAAB00000000	
Diptera	*Anopheles koliensis*	JXXB01000001	
Diptera	*Anopheles maculatus*	KZ061539	
Diptera	*Anopheles melas*	KI919284	
Diptera	*Anopheles merus*	KI915156	
Diptera	*Anopheles minimus*	KB663610	
Diptera	*Anopheles nili*	ATLZ01000001	
Diptera	*Anopheles punctulatus*	JXXA01000001	
Diptera	*Anopheles quadriannulatus*	KB665398	
Diptera	*Anopheles sinensis*	KL639024	
Diptera	*Anopheles stephensi*	KE388890	
Coleoptera	*Anoplophora glabripennis*	KB933500	
Lepidoptera	*Antheraea yamamai*	PRJNA383008	GigaDB Database
Hymenoptera	*Aphaenogaster ashmeadi*	NJRQ01000001	
Hymenoptera	*Aphaenogaster floridana*	NJRP01000001	
Hymenoptera	*Aphaenogaster fulva*	NJRO01000001	
Hymenoptera	*Aphaenogaster miamiana*	NJRN01000001	
Hymenoptera	*Aphaenogaster picea*	NJRM01000001	
Hymenoptera	*Aphaenogaster rudis*	NJRL01000001	
Hymenoptera	*Apis cerana*	NW_016017455	
Hymenoptera	*Apis dorsata*	NW_006263304	
Hymenoptera	*Apis florea*	NW_003789104	
Hymenoptera	*Apis mellifera*	NC_037638 - NC_037653	1 dna:chromosome_group chromosome_group:Amel_4.5:1:1:29893408:1 REF
Hymenoptera	*Athalia rosae*	NW_012161901	
Hymenoptera	*Atta cephalotes*	GCF_000143395	>scaffold00001 dna:supercontig supercontig:Attacep1.0:scaffold00001:1:15679321:1 REF
Hymenoptera	*Atta colombica*	NW_017258044	

Order	Species	Accession	Notes
Diptera	*Bactrocera dorsalis*	NW_011869236	
Diptera	*Bactrocera latifrons*	NW_017534406	
Diptera	*Bactrocera oleae*	NW_013581209	
Diptera	*Bactrocera tryoni*	JHQJ01000001	
Ephemeroptera	*Baetis rhodani*	LVVX01000001	
Diptera	*Belgica antarctica*	JPYR01000001	
Hemiptera	*Bemisia tabaci*	NW_017547088	
Lepidoptera	*Bicyclus anynana*	NW_019862748	
Blattodea	*Blattella germanica*	PYGN01000001	
Hymenoptera	*Bombus impatiens*	JH157811	
Hymenoptera	*Bombus terrestris*	CM001169 - CM001186	>B01 dna:chromosome_group chromosome_group:Bter_1.0:B01:1:17153651:1 REF
Lepidoptera	*Bombyx huttoni*	ASCI01000001	
Lepidoptera	*Bombyx mori*	GCF_000151625	>scaf1 dna:supercontig supercontig:ASM15162v1:scaf1:1:16203812:1 REF
Lepidoptera	*Calephelis nemesis*	NJDD01000001	
Lepidoptera	*Calephelis virginiensis*	NJDC01000001	
Diptera	*Calliphora vicina*	JXOT01000001	
Odonata	*Calopteryx splendens*	LYUA01000001	
Lepidoptera	*Calycopis cecrops*	LUGF01000001	
Hymenoptera	*Camponotus floridanus*	NW_020229214	
Diplura	*Catajapyx aquilonaris*	KZ537955	
Hymenoptera	*Cecidostiba fungosa*	UCOJ01000001	
Hymenoptera	*Cecidostiba semifascia*	UCQR01000001	
Isoptera	*CEME01 microsat.txt*	CEME01000001	
Hymenoptera	*Cephus cinctus*	NW_014332639	
Hymenoptera	*Ceratina calcarata*	NW_017130860	
Diptera	*Ceratitis capitata*	NW_019376232	
Hymenoptera	*Ceratosolen solmsi*	NW_011948412	

Order	Species	Accession	Notes
Diptera	*Chaoborus trivitattus*	JXOU01000001	
Lepidoptera	*Chilo suppressalis*	ANCD01000001	
Diptera	*Chironomus riparius*	JXPV01000001	
Diptera	*Chironomus tentans*	HG428765	
Hemiptera	*Cimex hemipterus*	LRFB01000001	
Hemiptera	*Cimex lectularius*	NW_019942502	
Diptera	*Cirrula hians*	JXOS01000001	
Phasmatodea	*Clitarchus hookeri*	NQII01000001	
Diptera	*Clogmia albipunctata*	JXOV01000001	
Diptera	*Clunio marinus*	CVRI01000001	
Diptera	*Coboldia fuscipes*	JXOR01001678	
Diptera	*Condylostylus patibulatus*	JXOW01000001	
Hymenoptera	*Copidosoma floridanum*	NW_019379452	
Hymenoptera	*Cotesia vestalis*	JZSA01000001	
Blattodea	*Cryptotermes secundus*	NW_019718022	
Diptera	*Culex quinquefasciatus*	NZ_AAWU00000000.1	>supercont3.1 dna:supercontig supercontig:CpipJ2:supercont3.1:1:3873040:1 REF
Diptera	*Culicoides sonorensis*	OGVF02000001	
Hymenoptera	*Cyphomyrmex costatus*	NW_017265214	
Hemiptera	*Dactylopius coccus*	JMCM01001127	
Lepidoptera	*Danaus plexippus*	DPSCF300001	
Coleoptera	*Dendroctonus ponderosae*	APGK00000000.1 9 (INSDC)	>Seq_1103023 dna:supercontig supercontig:DendPond_male_1.0:Seq_1103023:1:4163268:1 REF
Coleoptera	*Diabrotica virgifera*	KZ688668	
Hymenoptera	*Diachasma alloeum*	NW_015144998	
Hemiptera	*Diaphorina citri*	NW_007377440	
Hymenoptera	*Dinoponera quadriceps*	NW_014554123	
Hymenoptera	*Diuraphis noxia*	NW_015368242	
Diptera	*Drosophila albomicans*	JH838198	

Order	Species	Accession	Notes
Diptera	*Drosophila americana*	CWKB01000001	
Diptera	*Drosophila ananassae*	QMES00000000.2 (GenBank)	>scaffold_13340 dna:supercontig supercontig:dana_caf1:scaffold_13340:1:23697760:1 REF
Diptera	*Drosophila arizonae*	NW_017127682	
Diptera	*Drosophila biarmipes*	NW_016028565	
Diptera	*Drosophila bipectinata*	NW_016083910	
Diptera	*Drosophila busckii*	NC_030802	
Diptera	*Drosophila elegans*	NW_016078481	
Diptera	*Drosophila erecta*	QMER00000000.2 (GenBank)	>scaffold_4929 dna:supercontig supercontig:dere_caf1:scaffold_4929:1:26641161:1 REF
Diptera	*Drosophila eugracilis*	NW_016073535	
Diptera	*Drosophila ficusphila*	NW_016067781	
Diptera	*Drosophila grimshawi*	GCF_000005155.2	>scaffold_15110 dna:supercontig supercontig:dgri_caf1:scaffold_15110:1:24565398:1 REF
Diptera	*Drosophila hydei*	NW_019378586	
Diptera	*Drosophila kikkawai*	NW_016062640	
Diptera	*Drosophila melanogaster*	GCF_000001625.3	>2L dna:chromosome chromosome:BDGP6:2L:1:23513712:1 REF
Diptera	*Drosophila miranda*	NC_030302.1	
Diptera	*Drosophila mojavensis*	GCF_000005175.2	>scaffold_6540 dna:supercontig supercontig:dmoj_caf1:scaffold_6540:1:34148556:1 REF
Diptera	*Drosophila montana*	LUVX01000001	
Diptera	*Drosophila nasuta*	LYTC01000001	
Diptera	*Drosophila navojoa*	NW_017181428	
Diptera	*Drosophila novamexicana*	QMEP01000001	
Diptera	*Drosophila obscura*	NW_019152098	
Diptera	*Drosophila persimilis*	GCF_003286085.1	>scaffold_0 dna:supercontig supercontig:dper_caf1:scaffold_0:1:11822988:1 REF
Diptera	*Drosophila pseudoobscura*	GCF_000001765.3	>2 dna:chromosome chromosome:Dpse_3.0:2:1:30819483:1 REF
Diptera	*Drosophila rhopaloa*	NW_016039821	

Order	Species	Accession	Notes
Diptera	*Drosophila sechellia*	GCF_004382195.1	>scaffold_0 dna:supercontig supercontig:dsec_caf1:scaffold_0:1:21120651:1 REF
Diptera	*Drosophila serrata*	NW_018366212	
Diptera	*Drosophila simulans*	GCF_000754195.2	>2L dna:chromosome chromosome:ASM75419v3:2L:1:23539531:1 REF
Diptera	*Drosophila subobscura*	NGKO01001875	
Diptera	*Drosophila suzukii*	NW_016019885	
Diptera	*Drosophila takahashii*	NW_016034088	
Diptera	*Drosophila virilis*	GCF_003285735.1	>scaffold_13049 dna:supercontig supercontig:dvir_caf1:scaffold_13049:1:25233164:1 REF
Diptera	*Drosophila willistoni*	GCF_000005925.1	>scf2_1100000004943 dna:supercontig supercontig:dwil_caf1:scf2_1100000004943:1:16660200:1 REF
Diptera	*Drosophila yakuba*	GCF_000005975.2	>2L dna:chromosome chromosome:dyak_caf1:2L:1:22324452:1 REF
Phasmatodea	*Dryococelus australis*	NKXL01000001	
Hymenoptera	*Dufourea novaeangliae*	NW_015373878	
Ephemeroptera	*Ephemera danica*	KZ497563	
Diptera	*Ephydra gracilis*	JXPQ01000001	
Diptera	*Eristalis dimidiata*	JXPC01000001	
Hymenoptera	*Eufriesea mexicana*	NW_016907855	
Hymenoptera	*Euglossa Dilemma*	NIJG01000001	
Hymenoptera	*Eupelmus annulatus*	UDEW01000001	
Hymenoptera	*Eupelmus urozonus*	UELX01000001	
Diptera	*Eutreta diana*	JXPB01000001	
Hemiptera	*Ferrisia virgata*	FIZR01000001	
Diptera	*Fopius arisanus*	NW_011887740	
Thysanoptera	*Frankliniella occidentalis*	KZ555990	
Lepidoptera	*Galleria mellonella*	NTHM01000001	
Hemiptera	*Gerris buenoi*	KZ651037	
Diptera	*Glossina austeni*	KK502400	
Diptera	*Glossina brevipalpis*	KK351011	

Order	Species	Accession	Notes
Diptera	*Glossina fuscipes*	GCA_000671735.1	>Scaffold0 dna:supercontig supercontig:GfusI1:Scaffold0:1:3329503:1
Diptera	*Glossina morsitans*	GCA_001014515.1	>scf7180000652170 dna:supercontig supercontig:GmorY1:scf7180000652170:1:25362821:1
Diptera	*Glossina pallidipes*	GCA_000688715.1	>Scaffold0 dna:supercontig supercontig:GpallI1:Scaffold0:1:5836930:1
Diptera	*Glossina palpalis*	KN796171	
Hymenoptera	*Goniozus legneri*	NCVS01000001	
Hymenoptera	*Habropoda laboriosa*	NW_017100116	
Diptera	*Haematobia irritans*	PGFW01000001	
Hemiptera	*Halyomorpha halys*	NW_020110170	
Hymenoptera	*Harpergnathos saltator*	NW_020229871	
Lepidoptera	*Heliconius cydno*	FAPN01000001	
Lepidoptera	*Heliconius elevatus*	FAPS01000001	
Lepidoptera	*Heliconius ethilla*	FAPJ01000001	
Lepidoptera	*Heliconius hecale*	FAPG01000001	
Lepidoptera	*Heliconius hecuba*	FAQH01000001	
Lepidoptera	*Heliconius heurippa*	FAPY01000001	
Lepidoptera	*Heliconius hierax*	FAQL01000001	
Lepidoptera	*Heliconius ismenius*	FAPP01000001	
Lepidoptera	*Heliconius melpomene*	HE671591	
Lepidoptera	*Heliconius numata*	FASQ01000001	
Lepidoptera	*Heliconius pachinus*	FASS01000001	
Lepidoptera	*Heliconius pardalinus*	FASU01000001	
Lepidoptera	*Heliconius timareta*	FAUB01000001	
Lepidoptera	*Heliconius wallacei*	FAUM01000001	
Lepidoptera	*Heliconius xanthocles*	FAUE01000001	
Lepidoptera	*Helicoverpa armigera*	NW_018395390	
Lepidoptera	*Helicoverpa punctigera*	Hpuns12202012	Accessed from i5k project

Order	Species	Accession	Notes
Lepidoptera	*Helicoverpa zea*	KZ116068	
Lepidoptera	*Heliothis virescens*	NWSH01000001	
Diptera	*Hermetia illucens*	JXPW01000001	
Diptera	*Holcocephala fusca*	JXPE01000001	
Hemiptera	*Homalodisca vitripennis*	KZ826510	
Coleoptera	*Hypothenemus hampei*	LBGY01000001	
Plecoptera	*Isoperla grammatica*	LVVW010000001	
Lepidoptera	*Laparus doris*	FAQD01000001	
Hymenoptera	*Lasioglossum albipes*	ANOB01000001	
Hymenoptera	*Lasius niger*	LBMM01000001	
Orthoptera	*Laupala kohalensis*	NNCF01000001	
Plecoptera	*Lednia tumana*	QKMV01000001	
Hymenoptera	*Lepidotrigona ventralis*	NIPQ01000001	
Lepidoptera	*Leptidea sinapsis*	FZQP01000001	
Coleoptera	*Leptinotarsa decemlineata*	NW_019289415	
Hymenoptera	*Leptopilina boulardi*	PQAT01000001	
Hymenoptera	*Leptopilina clavipes*	JUFY01000001	
Lepidoptera	*Lerema accius*	LGAG01000001	
Odonata	*Libellula fulva*	GCA_000376725.2	
Trichoptera	*Limnephilus lunatus*	KZ319399	
Hymenoptera	*Linepithema hunile*	NW_012157838	
Diptera	*Liriomyza trifolii*	JXHJ01000001	
Orthoptera	*Locusta migratoria*	AVCP010000001	
Diptera	*Lucilia cuprina*	JRES01001160	ref
Diptera	*Lucilia sericata*	JXPF01000001	
Diptera	*Lutzomyia longipalpis*	JH689321	
Archaeognatha	*Machilis hrabei*	GCA_003456935.1	

Order	Species	Accession	Notes
Hemiptera	*Maconellicoccus hirsutus*	FIZS01000001	
Hymenoptera	*Macrocentrus cingulum*	MVJL01000001	
Lepidoptera	*Mamestra configurata*	NDFZ01000001	
Lepidoptera	*Manduca sexta*	JH668279	ref
Diptera	*Mayetiola destructor*	GL501517	ref
Phasmatodea	*Medauroidea extradentata*	PNEQ01000001	
Hymenoptera	*Megachile rotundata*	NW_003797049	
Diptera	*Megaselia abdita*	JXPG01000001	
Diptera	*Megaselia scalaris*	GCA_000341915.2	>scaffold9042 dna:scaffold scaffold:Msca1:scaffold9042:1:114435:1 REF
Hymenoptera	*Megastigmus dorsalis*	UELU01000001	
Hymenoptera	*Megastigmus stigmatizans*	UELV01000001	
Hemiptera	*Melanaphis sacchari*	NW_020270366	
Hymenoptera	*Melipona quadrifasciata*	KQ435686	
Lepidoptera	*Melitaea cinxia*	GCA_000716385.1	>scaffold1689 dna:scaffold scaffold:MelCinx1.0:scaffold1689:1:668473:1 REF
Strepsiptera	*Mengenill moldrzyki*	AGDA01000001	
Hymenoptera	*Microplitis demolitor*	NW_014463221	
Diptera	*Mochlonyx cinctipes*	JXPH01000001	
Hymenoptera	*Monomorium pharaonis*	NW_012205351	
Diptera	*Musca domestica*	NW_004754939	
Hemiptera	*Myzus persicae*	NW_019100468	
Hymenoptera	*Nasonia giraulti*	GL273804	
Hymenoptera	*Nasonia longicornis*	GL277950	
Hymenoptera	*Nasonia vitripennis*	GCF_009193385.2	ref>1 dna:chromosome chromosome:Nvit_2.1:1:1:33571687:1 REF
Diptera	*Neobellieria bullata*	JXPI01000001	
Hymenoptera	*Neodiprion lecontei*	NW_015380904	
Lepidoptera	*Neruda aoede*	FAPZ01000001	

Order	Species	Accession	Notes
Coleoptera	*Nicrophorus vespilloides*	NW_017095466	
Hemiptera	*Nilaparvata lugens*	NW_019105540	
Hemiptera	*Oncopeltus fasciatus*	KZ806866	
Coleoptera	*Onthophagus taurus*	NW_019280316	
Hymenoptera	*Ooceraea biroi*	NW_011924876	
Lepidoptera	*Operophtera brumata*	JTDY01000001	
Hymenoptera	*Ormyrus nitidulus*	UCOL01000001	
Hymenoptera	*Ormyrus pomaceus*	UCOM01000001	
Hymenoptera	*Orussus abietinus*	NW_019394231	
Coleoptera	*Oryctes borbonicus*	LJIG01000001	
Hemiptera	*Pachypsylla venusta*	KZ545295	
Lepidoptera	*Papilio glaucus*	JWHW01000001	
Lepidoptera	*Papilio machaon*	NW_014478584	
Lepidoptera	*Papilio memnon*	BDMF01000001	
Lepidoptera	*Papilio polytes*	NW_013524711	
Lepidoptera	*Papilio xuthus*	NW_013528584	
Hemiptera	*Paracoccus marginatus*	FIZT01000001	
Diptera	*Paykullia maculata*	NDXZ01000001	
Pthiraptera	*Pediculus humanus*	DS235882	
Blatoodea	*Periplaneta americana*	PGRX01000001	
Hemiptera	*Philaenus spumarius*	MPAX010000001	
Diptera	*Phlebotomus papatasi*	JH660948	
Lepidoptera	*Phoebis sennae*	LQNK01000631	
Diptera	*Phormia regina*	MINK01000001	
Diptera	*Phortica variegata*	JXPM01000001	
Lepidoptera	*Pieris rapae*	NW_019093120	
Hemiptera	*Piezodorus guildinii*	JTEQ01000001	
Lepidoptera	*Plodia interpunctella*	LT849405	

Order	Species	Accession	Notes
Lepidoptera	*Plutella xylostella*	NW_011952010	
Hymenoptera	*Pogomyrmex barbatus*	NW_011929472	
Coleoptera	*Pogonus chalceus*	CM008230	
Hymenoptera	*Polistes canadensis*	NW_014569547	
Hymenoptera	*Polistes dominula*	NW_015148970	
Coleoptera	*Priacma serrata*	AGRH01000001	
Diptera	*Proctacanthus coquilletti*	MNCL01000001	
Hemiptera	*Pseudococcus longispinus*	FIZU01000001	
Hymenoptera	*Pseudomyrmex gracilis*	NW_018020584	
Diptera	*Rhagoletis zephyria*	NW_016157085	
Hemiptera	*Rhodnius prolixus*	KQ034056	
Diptera	*Scaptodrosophila lebanonesis*	QMEN01000001	
Hemiptera	*Schizaphis graminum*	QEWZ01000001	
Hemiptera	*Sipha flava*	NW_020271713	
Coleoptera	*Sitophilus oryzae*	PPTJ01000001	
Hymenoptera	*Solenopsis invicta*	GCF_000188075.2	>Si_gnG.scaffold02694 dna:supercontig supercontig:Si_gnG:Si_gnG.scaffold02694:1:6355204:1 REF
Diptera	*Sphyracephala brevicornis*	JXPL01000001	
Lepidoptera	*Spodoptera frugiperda*	NJHR01000001	
Lepidoptera	*Spodoptera litura*	NC_036187	
Diptera	*Stomoxys calcitrans*	NW_013171869	
Hymenoptera	*Synergus umbraculus*	UCOK01000001	
Diptera	*Teleopsis dalmanni*	NLCU01024205	
Hymenoptera	*Temnothorax curvispinosus*	NW_020209781	
Diptera	*Tephritis californica*	JXPN01000001	
Diptera	*Themira minor*	JXPZ01000001	
Phasmatodea	*Timema cristinae*	CM009472	

Order	Species	Accession	Notes
Diptera	*Tipula oleracea*	JXPP01000001	
Hemiptera	*Trabutina mannipara*	FKYK01000001	
Hymenoptera	*Trachymyrmex cornetzi*	NW_017280593	
Hymenoptera	*Trachymyrmex septentrionalis*	NW_017300354	
Hymenoptera	*Trachymyrmex zeteki*	NW_017253421	
Coleoptera	*Tribolium castaneum*	GCF_000002335.3	>LGX dna:chromosome_group chromosome_group:Tcas5.2:LGX:1:8676460:1 REF
Hymenoptera	*Trichogramma pretiosum*	NW_019640787	
Hymenoptera	*Trichomalopsis sarcophagae*	NNAY01000001	
Lepidoptera	*Trichoplusia ni*	GCF_003590095.1	
Hemiptera	*Trionymus perrisii*	FIZV01000001	
Diptera	*Trupanea jonesi*	JXQA01000001	
Lepidoptera	*Vanessa tameamea*	PEHJ01000001	
Hymenoptera	*Vollenhovia emeryi*	NW_011953989	
Hymenoptera	*Wasmannia auropunctata*	NW_012020309	
Orthoptera	*Xenocatantops brachycerus*	OFSG01000001	
Diptera	*Zaprionus indianus*	LWKS01000001	
Diptera	*Zeugodacus cucurbitae*	NW_011863665	
Blattodea	*Zootermopsis nevadensis*	GCF_000696155.1	>scaffold24 dna:supercontig supercontig:ZooNev1.0:scaffold24:1:5111804:1 REF

Supplemental Table 2. Species and gene sequences used for construction of the phylogenetic tree. Accession numbers are included for each of the 8 genes (argk, COI, COII, cytb, ef1a, 12S, 18S, 28S). Only inclusive of those species which met the BUSCO score threshold (0.9 or 90%)

Species	Argk	COI	COII	Cytb	Ef1a	12S	18S	28S
Achalarus lyciades		MG646077.1						
Acromyrmex echinatior	XM_011066862.1							
Acyrthosiphon pisum	XM_008187305.2	AB470712.1	FJ982392.1	FJ752022.1	FJ982418.1	JX965977.1	U27819.1	
Aedes aegypti	XM_001657339.2	KX420491.1	KC913582.1	AJ970959.1	KY000702.1	AY356400.1	U65375.1	
Aedes albopictus	XM_019692640.1	MF767287.1	AF324898.1	AJ971002.1		AF034471.1	HQ010437.1	
Aethina tumida	XM_020016979.1	KU179767.1	KC966688.1			KP133876.1	KP134008.1	
Agrilus planipennis	XM_018470050.2	GU013563.1		XM_018479674.2				XM_018468835.2
Aleochara bilineata		KM847308.1						
Amyelois transitella	XM_013328496.1							
Anopheles alnimanus								
Anopheles aquasalis		AF548902.1	U92375.1				AF417769.1	AF417804.1
Anopheles arabiensis		DQ465294.1	AF417741.1					
Anopheles atroparvus	GQ906807.1	KU877020.1					AM072973.1	LK054523.1
Anopheles christyi								
Anopheles coluzzii		MH025880.1						
Anopheles cracens		DQ897948.1						
Anopheles culicifacies		MH512896.1	EF208913.1	KP197030.1		KP893744.1		DQ173155.1
Anopheles darlingi		KP193458.1	U92383.1				AF417770.1	AF417805.1
Anopheles dirus		DQ897946.1	JX070696.1	KF431914.1			AF417779.1	AF417814.1
Anopheles epiroticus		AY789202.1						
Anopheles farauti		KF202472.1	DQ674734.1	HQ840893.1		AF121065.1	AF121054.1	AF417815.1
Anopheles funestus		KU380404.1	AJ512749.1	DQ287361.1		DQ287369.1	AF417780.1	AF417816.1
Anopheles gambiae		DQ465336.1	AF417742.1			U35784.1	AM157179.1	KC177663.1
Anopheles koliensis		KU662306.1	U94304.1	HQ840920.1		AF121072.1	AF121061.1	
Anopheles maculatus		EU256336.1	EF208910.1				AF440198.1	AY120851.1
Anopheles melas						U35794.1		
Anopheles merus						U35791.1		
Anopheles minimus		KU497613.1	JQ042279.1	KF431913.1		AF293664.1	AF417781.1	DQ523567.1
Anopheles quadriannulatus		KR014849.1				U35792.1		
Anopheles sinensis		MG028766.1	KX840670.1	MG028854.1		AF293657.1		KJ617054.1
Anopheles stephensi		MF124611.1	DQ026675.1	AF311254.1		AF034465.1	AF417784.1	AF417820.1
Anoplophora glabripennis	XM_018717621.1	AB439154.1				KF737699.1	KF142012.1	
Antheraea yamamai		GU707195.1				AY037835.1		
Aphaenogaster ashmeadi		KP730068.1						
Aphaenogaster floridana		KP730073.1						
Aphaenogaster fulva		KP730082.1						
Aphaenogaster miamiana		KP730088.1						
Aphaenogaster picea		KP730106.1						
Aphaenogaster rudis	FJ824372.1	MG341765.1		EU658912.1			FJ824191.1	FJ824281.1

Species	Argk	COI	COII	Cytb	Ef1a	12S	18S	28S
Apis cerana	XM_017049717.1	KT960839.1		EF467437.1			HM750231.1	
Apis dorsata	XM_006609081.1	KU752355.1	AF153126.1	KP259252.1			HM750230.1	
Apis florea	XM_012489495.1	AB284150.1	MG548267.1	KP259265.1			HQ108053.1	
Apis mellifera	AF023619.1	JN293758.1		EU513289.1			KC413715.1	
Athalia rosae	XM_012404176.2	KF642888.1		KF528568.1			AB064266.1	
Atta cephalotes	XM_012202340.1	MG948895.1						
Atta colombica	XM_018192232.1	EU847939.1	EU448260.1	KF500036.1				
Bactrocera dorsalis	XM_011214398.2	KX051723.1	EU926791.1	JF521166.1	GU339154.1		U01251.1	
Bactrocera latifrons	XM_018934259.1	MF140511.1	AB687549.1		MG683593.1			
Bactrocera oleae	XM_014233915.1	KY111527.1			MG683538.1	AY037337.1		
Bactrocera tryoni		MH135262.1	MG020808.1	JQ420932.1	MG683620.1	AY037345.1		
Belgica antarctica		JQ672717.1					DQ507305.1	DQ459549.1
Bemisia tabaci	XM_019046375.1	MF289534.1						
Bicyclus anynana	XM_024078988.1	KR139591.1	AY040163.1		KM923784.1			
Blattella germanica	FJ855501.1	S72627.1	DQ874268.1	KY014629.1		AY536379.1	FJ806322.1	
Bombus impatiens	XM_012391881.2	GU806825.1		AF281169.1				
Bombus terrestris	XM_003401454.3	JQ843670.1	X90406.1	KP670308.1				
Bombyx mori	XM_021351952.1	AB649195.1		AY343547.1	NM_001044045.1	AY037827.1	KF982847.1	
Calephelis nemesis								
Calephelis virginiensis								
Calopteryx splendens		DQ411707.1					DQ008208.1	
Calycopis cecrops		GU089704.1						
Camponotus floridanus	XM_025406946.1	AF186362.1						
Catajapyx aquilonaris							EU368600.1	EF199978.2
Cecidostiba fungosa		HM574094.1		JQ417157.1				
Cecidostiba semifascia		HM574093.1						
Cephus cinctus	XM_015732313.2	HM432906.1		XM_015752756.2				
Ceratina calcarata	XM_018034016.2	KJ166268.1		GU321567.1			AY995679.1	
Ceratitis capitata	XM_004526740.3	MG764540.1	DQ011889.1	DQ006914.1	MG683583.1		KC177300.1	
Ceratosolen solmsi	XM_011506811.1	AY842421.1						
Chironomus riparius		KR756187.1					DQ657920.1	AY820919.1
Chironomus tentans		KY225374.1		AF109712.1				
Cimex lectularius	XM_014383694.2	KR044731.1		XM_014405864.1			KJ461298.1	
Clitarchus hookeri		GU194457.1	KY263955.1					
Clunio marinus		LN851838.1						
Coboldia fuscipes		MG296087.1		KC177601.1		KC177484.1	KC177282.1	KC177651.1
Copidosoma floridanum	XM_014364357.2	KR877543.1					KF850043.1	
Cotesia vestalis		KY832157.1		KX604901.1	KX605227.1		JX399880.1	AJ535953.1
Cryptotermes secundus	XM_023849251.1		EF442718.1	AF189120.1		DQ441676.1	DQ882635.1	
Culex quinquefasciatus	XM_001842457.1	KR768063.1	JQ716608.1	HQ709409.1				HM807289.1
Culicoides sonorensis		KR683671.1			KP310110.1			
Cyphomyrmex costatus	XM_018545298.1							
Danaus plexippus	EU141261.1	KT133515.1	KX110382.1	KP007541.1	DQ157894.1	AF392051.1	AF454921.1	GQ229491.1
Dendroctonus ponderosae	XM_019909387.1	JF888167.1					KJ531053.1	
Diabrotica virgifera	XM_028298329.1	MH323083.1				AY533603.1		AY243734.2
Diachasma alloeum	XM_015265737.1	KR890748.1						

Species	Argk	COI	COII	Cytb	Ef1a	12S	18S	28S
Dinoponera quadriceps	XM_014611839.1							
Drosophila americana		JN019935.1	AY646740.1	GU299316.1			EU188739.1	JF735897.1
Drosophila ananassae		MG557557.1	KM487042.1	EU601719.1		EU189427.1	XR_046314.1	JF735891.1
Drosophila arizonae	XM_018018451.1	MF443102.1	DQ436072.1					
Drosophila biarmipes	XM_017097033.1	AY098456.1	AF474094.1					
Drosophila bipectinata	XM_017247855.1	AB032131.1	GQ376042.1	KM487044.1				
Drosophila busckii	XM_017991261.1	MF882465.1	KF601930.1					
Drosophila elegans	XM_017266287.1	AB032130.1	AF461307.1					
Drosophila erecta	XM_001971531.3	KX771108.1	GQ244453.1	JX500968.1				
Drosophila eugracilis	XM_017228296.1	AY098461.1	AF474079.1					
Drosophila ficusphila	XM_017198069.1	AB032133.1	AY757273.1					
Drosophila grimshawi		GU597459.1	GU597491.1			EU189429.1		GU597390.1
Drosophila hydei	XM_023320454.1	MG086288.1	AF145888.1					
Drosophila kikkawai	XM_017176053.1	KY973965.1	KP731163.1					
Drosophila melanogaster	U26939.1	KC750827.1	GQ376046.1	AM403328.1	NM_079872.4	X97155.1	NR_133559.1	NR_133562.1
Drosophila miranda	XM_017285861.1	U51608.1	M95148.1	EF216276.1				XM_017287623.1
Drosophila mojavensis		DQ383730.1	AY437281.1	EU494122.1		EU494550.1	XR_047783.1	GQ244442.1
Drosophila montana		MG080424.1	DQ426799.1					JF735907.1
Drosophila nasuta		GU597466.1	AB932783.1	EU494105.1		AF332704.1		GU597436.1
Drosophila novamexicana		JF735929.1	JF735934.1	AY646768.1				AF184036.1
Drosophila obscura	XM_022363945.1	U51614.1	AF081356.1	EF216277.1				XM_022368481.1
Drosophila persimilis	XM_002016998.2	AF451101.1	M95143.1	EF216278.1				XM_026990083.1
Drosophila pseudoobscura		AF451073.1	M95150.1	EF216279.1		AF220088.1	XR_053284.1	AF184037.1
Drosophila rhopaloa	XM_017113570.1							XM_017136819.1
Drosophila sechellia		KJ426007.1	GQ244458.1				XR_048770.1	
Drosophila serrata	XM_020962843.1	HQ631573.1	GQ376043.1	XM_020948685.1				
Drosophila simulans		KT112992.1	GQ222022.1				AY037174.1	HQ110541.1
Drosophila subobscura		MG082253.1	AF187689.1	EF216282.1		AF220094.1		EF216305.1
Drosophila suzukii	XM_017078652.1	AB032128.1	LN867083.1					
Drosophila takahashii	XM_017144515.1	JF735911.1	AF474089.1					
Drosophila virilis		MH423345.1	HQ110559.1	AY646772.1		AY560540.1	XR_049301.1	AF184035.1
Drosophila willistoni	XM_023178113.1	KT194321.1	HQ110560.1	EU494155.1		X97154.1		
Drosophila yakuba			DQ382818.1				M26990.1	HQ110545.1
Dufourea novaeangliae	XM_015577015.1	HM414187.1						
Ephemera danica		KY261917.1	KF855909.1			KF855788.1	KF855833.1	KF855809.1
Ephydra gracilis								
Eufriesea mexicana	XM_017898377.1							
Euglossa Dilemma								
Eupelmus annulatus		MG343741.1		KR348775.1				
Eupelmus urozonus		KR348766.1		KR348814.1				
Ferrisia virgata		KJ530607.1			LC278436.1	HQ893809.1	AY426079.1	LC278437.1
Fopius arisanus	XM_011304607.1	KY689110.1		KC753514.1		KC594927.1		
Frankliniella occidentalis	MH319815.1	MG365569.1	KY775411.1	KJ916245.1	GU148015.1		JX002704.1	XM_026437035.1
Galleria mellonella	XM_026900087.1	MK187421.1	M83967.1				AF286298.1	
Gerris buenoi		MG406973.1	KX821870.1	KX821900.1		KX821796.1	KX821825.1	KX821840.1
Glossina austeni		EU591834.1						EF531133.1

Species	Argk	COI	COII	Cytb	Ef1a	12S	18S	28S
Glossina brevipalpis		EU591869.1				MK034891.1		EF531134.1
Glossina fuscipes		MK094173.1		EU562281.1		MK034887.1		
Glossina morsitans		EF531200.1	KR820741.1	KC177594.1		MK034895.1	KC177312.1	KC177834.1
Glossina pallidipes		HQ702375.1				MK034890.1		EF531136.1
Glossina palpalis		KF597291.1		KJ013528.1		MK034896.1	AF322431.1	EF531137.1
Goniozus legneri								GU213948.1
Habropoda laboriosa	XM_017938075.1	KY321466.1						
Halyomorpha halys	XM_014425440.2	KY570297.1	KY710681.1	KF273412.1				
Harpergnathos saltator	XM_011149430.3							
Heliconius melpomene	KP073002.1	EU449025.1			DQ448447.1			
Helicoverpa armigera	JN185455.1	KP210095.1	HQ677778.1	EF410045.1		AF389421.1	KT945996.1	
Helicoverpa punctigera		KX422509.1		EF410058.1				
Helicoverpa zea	HM068068.1	GU438926.1	HQ677777.1	HQ177612.1		HQ178018.1	KT946005.1	
Hypothenemus hampei		KX818264.1						
Lasioglossum albipes		GU706031.1						
Lasius niger	EU142995.1	MG344523.1	MF993323.1	JQ681115.1			KT443340.1	
Lepidotrigona ventralis	DQ813047.1							FJ042126.1
Leptidea sinapsis								
Leptinotarsa decemlineata	XM_023158001.1	AY165708.1						
Leptopilina boulardi		KY077405.1						
Leptopilina clavipes		KR899847.1						
Lerema accius		GU089979.1						
Libellula fulva		AF195745.1					DQ008205.1	FJ596602.1
Linepithema hunile	XM_012359713.1	FJ161727.1		EF363097.1			AH012894.2	
Lucilia cuprina	XM_023444944.1	KX053871.1	DQ345111.1	DQ407477.1				
Maconellicoccus hirsutus		KY373171.1			KY678264.1		AY426033.1	AY427403.1
Macrocentrus cingulum		JF963516.1		HQ177822.1	HQ177281.1			
Manduca sexta		JN678236.1					U88190.1	
Mayetiola destructor		MG130753.1		AF488423.1		MH282938.1	KJ917326.1	KC177649.1
Megachile rotundata	XM_012291469.1	MG351078.1						
Megastigmus dorsalis		AY317240.1		AY898702.1			AY317197.1	AY317161.1
Megastigmus stigmatizans				AY898703.1				
Melanaphis sacchari	XM_025334412.1	MG838315.1	KT376991.1					
Melipona quadrifasciata	EU163050.1	FJ975764.1		EF529493.1				
Microplitis demolitor	XM_008546537.2	HM904940.1			DQ538655.1			
Mochlonyx cinctipes								
Monomorium pharaonis	XM_012676742.2	KC419419.1					JQ695802.1	
Musca domestica	XM_011296089.2	MG091420.1	HM016775.1	DQ657064.1	DQ657113.1	AY573084.1	GQ465780.1	
Myzus persicae	XM_022308072.1	KY509874.1	AF143592.1	HQ528298.1	EF419315.1	JX965999.1		
Nasonia giraulti		EF638434.1						
Nasonia longicornis		FJ554529.1						
Nasonia vitripennis	XM_016989842.1	EF638430.1					GQ410677.1	
Neodiprion lecontei	XM_015666009.1			XM_015656300.1				
Nicrophorus vespilloides	XM_017930012.1	MH115515.1						EU147362.1
Nilaparvata lugens	XM_022337262.1	AB325705.1		JX556844.1		HQ116528.1	JF773148.1	
Oncopeltus fasciatus		MG406644.1	M83959.1				U15188.1	LN623664.1

Species	Argk	COI	COII	Cytb	Ef1a	12S	18S	28S
Onthophagus taurus	XM_023061824.1	MH020533.1	EU162431.1	XM_023055227.1	KC294268.1			DQ430885.1
Ooceraea biroi	XM_011351977.3							
Operophtera brumata		MG466845.1				AF232887.1		
Ormyrus nitidulus		HM574027.1		JQ417107.1				
Ormyrus pomaceus		HM574022.1		JQ417171.1				
Orussus abietinus	XM_012429232.2	KC976945.1		KF528558.1		JF505477.1	GQ410604.1	
Oryctes borbonicus		KJ915151.1						
Papilio glaucus	EU141279.1	KM548369.1						
Papilio machaon	XM_014507128.1	MK186611.1	LC189137.1		GU372637.1			
Papilio polytes	XM_013277807.1	HQ962338.1	LC189134.1	HM246439.1		GU196787.1		
Papilio xuthus	NM_001312616.1	JQ922032.1	LC189138.1	JQ924447.1	NM_001312698.1			
Paracoccus marginatus		KY373146.1					EU188580.1	AY427410.1
Paykullia maculata		MG967799.1		FJ025742.1	FJ025694.1	FJ025404.1	FJ025496.1	
Pediculus humanus		KX054334.1		GU323333.1	FJ267441.1	MH429017.1	AY077775.1	
Phoebis sennae		MF547337.1						
Phormia regina		GU013648.1	DQ345102.1	KF908169.1		AF262957.1		AF366685.1
Phortica variegata								
Pieris rapae	GU215936.1	JQ996397.1	GQ268364.1	JQ924443.1	GU372646.1	DQ150034.1	KC413860.1	
Plodia interpunctella	AJ315030.1	KT145708.1					KJ836335.2	
Plutella xylostella							JX390653.1	
Pogomyrmex barbatus	XM_011644610.2			AF202484.1				
Pogonus chalceus	JN170715.1	KU918208.1		KJ371137.1			GU556144.1	GU556114.1
Polistes canadensis	XM_014758359.1	AY663544.1						
Polistes dominula	XM_015332541.1	KR880422.1	KJ147264.1	KJ147292.1		GU596549.1		
Proctacanthus coquilletti		KT733476.1						
Pseudococcus longispinus		KY373111.1			JX677901.1	HQ893808.1	KT199023.1	KT199048.1
Pseudomyrmex gracilis	XM_020427193.1	KU985485.1					AY703529.1	
Rhagoletis zephyria	XM_017638032.1	MG825319.1	EU109172.1				U01267.1	
Rhodnius prolixus		AF449138.1		AF421339.1		AF394519.1	AJ421962.1	
Scaptodrosophila lebanonesis		EU493686.1	HQ110572.1	EU494188.1		EU494586.1		HQ110555.1
Schizaphis graminum		MG512288.1	AF469802.1	AJ315874.1		JX966014.1	AH003128.2	
Sipha flava	XM_025556687.1	KR044391.1	AF254094.1					
Sitophilus oryzae		KU494238.1	KX190772.1				AM946646.1	AY131070.1
Solenopsis invicta	XM_026137080.1	EF620560.1					AY334566.1	
Spodoptera frugiperda	KC262642.1	MF197868.1	HQ677792.1	HQ177674.1		HQ178084.1		
Spodoptera litura	XM_022978992.1	MG783870.1		HQ177695.1		HQ178113.1		
Stomoxys calcitrans	XM_013248543.1	MG097496.1	EU627744.1	EU851308.1	FJ025698.1	DQ656886.1	FJ025499.1	
Synergus umbraculus		DQ012655.1		GU386442.1				
Teleopsis dalmanni			KT186763.1	KC177592.1		KT186734.1	KC177302.1	KC177739.1
Temnothorax curvispinosus	MF436500.1	MG340300.1						XM_025036124.1
Timema cristinae		EU251511.1	JN615409.1					
Trabutina mannipara							EU188595.1	EU188518.1
Trachymyrmex cornetzi	XM_018522051.1	KT783624.1						XM_018521294.1

Species	Argk	COI	COII	Cytb	Ef1a	12S	18S	28S
Trachymyrmex septentrionalis	XM_018483182.1	EU561600.1						XM_018498567.1
Trachymyrmex zeteki	XM_018447315.1	KM224757.1						XM_018460735.1
Tribolium castaneum	XM_962718.3	KU913057.1		KJ003215.1	NM_001114363.1	KJ002951.1	KP419322.1	
Trichogramma pretiosum	XM_014382207.2	KM998973.1	AY676062.1	DQ181936.1				
Trichomalopsis sarcophagae								
Trichoplusia ni	XM_026882819.1	GU439174.1	HQ677815.1	AB125896.1			KY514086.1	
Vanessa tameamea	XM_026642261.1	HQ734891.1						
Vollenhovia emeryi	XM_012022523.1			MG255049.1			EF012949.1	XM_012025884.1
Wasmannia auropunctata	XM_011709607.1	MF926000.1		KX585845.1			EF012950.1	
Zaprionus indianus		EF632372.1	EF632396.1					GU597433.1
Zeugodacus cucurbitae		MH751503.1	MG020794.1		MG683520.1			
Zootermopsis nevadensis	XM_022061494.1	MF358687.1	GQ922444.1	EU253931.1		EU253721.1	EU253799.1	

Supplemental Table 3. Phylogeny fossil node constraints used for phylogeny construction with the means and 95% confidence intervals.

Node	Mean	2.5% Quantile	97.5% Quantile
Root	407.0	383	431
Hemiptera	289.0	232	346
Ephemeroptera/ Odonata	353.0	296	410
Hymenoptera	252.0	221	283
Diptera	157.0	108	206
Lepidoptera	147.0	116	178
Blattodea/ Phasmatodea	250.8	195	307
Coleoptera	258.0	246	270

Supplemental Table 4. P-values for ANOVAs for 2-6mer and total microsatellite content by order. While orders appeared to have significance with a standard ANOVA, all significance was removed when corrected for phylogenetic history. The p-values shown for the phylogenetically corrected ANOVA are the mean of the p-values calculated using each of the 100 trees from our posterior distribution. In no cases were any significant p-values found on the individual phylogenetically corrected ANOVAS from which these means were calculated.

Measure	Standard ANOVA	Phylogenetically corrected ANOVA
2mer	6.76E-6	0.867
3mer	9.58E-10	0.691
4mer	9.72E-8	0.782
5mer	0.005	0.964
6mer	9.72E-8	0.774
2-6mer	0.04E-2	0.932
bp/Mbp	0.487	0.999

Supplemental Table 5. P-values for ANOVAs for 2-6mer and total microsatellite content by centromere type. In no cases were monocentric and holocentric species significantly different. The p-values shown for the phylogenetically corrected ANOVA are the mean of the p-values calculated using each of the 100 trees from our posterior distribution. In no cases were any significant p-values found on the individual phylogenetically corrected ANOVAs from which these means were calculated.

Measure	Standard ANOVA	Phylogenetically corrected ANOVA
2mer	0.168	0.254
3mer	0.175	0.265
4mer	0.364	0.458
5mer	0.717	0.771
6mer	0.807	0.841
2-6mer	0.185	0.270
Bp/Mbp	0.109	0.187
